# A framework for evaluating the chemical knowledge and reasoning abilities of large language models against the expertise of chemists

**DOI:** 10.1038/s41557-025-01815-x

**Published:** 2025-05-20

**Authors:** Adrian Mirza, Nawaf Alampara, Sreekanth Kunchapu, Martiño Ríos-García, Benedict Emoekabu, Aswanth Krishnan, Tanya Gupta, Mara Schilling-Wilhelmi, Macjonathan Okereke, Anagha Aneesh, Mehrdad Asgari, Juliane Eberhardt, Amir Mohammad Elahi, Hani M. Elbeheiry, María Victoria Gil, Christina Glaubitz, Maximilian Greiner, Caroline T. Holick, Tim Hoffmann, Abdelrahman Ibrahim, Lea C. Klepsch, Yannik Köster, Fabian Alexander Kreth, Jakob Meyer, Santiago Miret, Jan Matthias Peschel, Michael Ringleb, Nicole C. Roesner, Johanna Schreiber, Ulrich S. Schubert, Leanne M. Stafast, A. D. Dinga Wonanke, Michael Pieler, Philippe Schwaller, Kevin Maik Jablonka

**Affiliations:** 1 Laboratory of Organic and Macromolecular Chemistry, Friedrich Schiller University Jena, Jena, Germany; 2 Helmholtz Institute for Polymers in Energy Applications Jena (HIPOLE Jena), Jena, Germany; 3 Institute of Carbon Science and Technology, CSIC, Oviedo, Spain; 4 QpiVolta Technologies Pvt Ltd, Bengaluru, India; 5 Laboratory of Artificial Chemical Intelligence, Institut des Sciences et Ingénierie Chimiques, École Polytechnique Fédérale de Lausanne, Lausanne, Switzerland; 6 National Centre of Competence in Research Catalysis, École Polytechnique Fédérale de Lausanne, Lausanne, Switzerland; 7 Department of Chemical Engineering and Biotechnology, University of Cambridge, Cambridge, UK; 8 Macromolecular Chemistry, University of Bayreuth, Bayreuth, Germany; 9 Laboratory of Molecular Simulation, Institut des Sciences et Ingénierie Chimiques, École Polytechnique Fédérale de Lausanne, Sion, Switzerland; 10 Institute for Inorganic and Analytical Chemistry, Friedrich Schiller University Jena, Jena, Germany; 11 Jena Center for Soft Matter, Friedrich Schiller University Jena, Jena, Germany; 12 Institute for Technical Chemistry and Environmental Chemistry, Friedrich Schiller University Jena, Jena, Germany; 13 Center for Energy and Environmental Chemistry Jena, Friedrich Schiller University Jena, Jena, Germany; 14 Intel Labs, Hillsboro, OR USA; 15 Theoretical Chemistry, Technische Universität Dresden, Dresden, Germany; 16 OpenBioML.org, London, UK; 17 Stability.AI, London, UK

**Keywords:** Cheminformatics, Chemical safety

## Abstract

Large language models (LLMs) have gained widespread interest owing to their ability to process human language and perform tasks on which they have not been explicitly trained. However, we possess only a limited systematic understanding of the chemical capabilities of LLMs, which would be required to improve models and mitigate potential harm. Here we introduce ChemBench, an automated framework for evaluating the chemical knowledge and reasoning abilities of state-of-the-art LLMs against the expertise of chemists. We curated more than 2,700 question–answer pairs, evaluated leading open- and closed-source LLMs and found that the best models, on average, outperformed the best human chemists in our study. However, the models struggle with some basic tasks and provide overconfident predictions. These findings reveal LLMs’ impressive chemical capabilities while emphasizing the need for further research to improve their safety and usefulness. They also suggest adapting chemistry education and show the value of benchmarking frameworks for evaluating LLMs in specific domains.

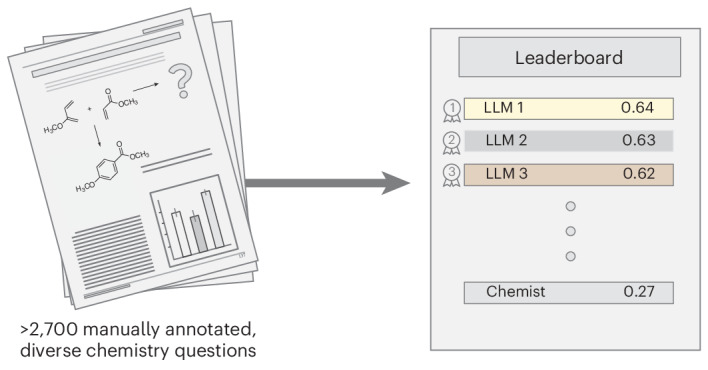

## Abstract (web summary)

Large language models are increasingly used for diverse tasks, yet we have limited insight into their understanding of chemistry. Now ChemBench—a benchmarking framework containing more than 2,700 question–answer pairs—has been developed to assess their chemical knowledge and reasoning, revealing that the best models surpass human chemists on average but struggle with some basic tasks.

## Main

Large language models (LLMs) are machine learning (ML) models trained on massive amounts of text to complete sentences. Aggressive scaling of these models has led to a rapid increase in their capabilities^1,2^, with the leading models now being able to pass the US Medical Licensing Examination^3^ or other professional licensing exams. They also have been shown to design and autonomously perform chemical reactions when augmented with external tools such as web search and synthesis planners^4–7^. While some see ‘sparks of artificial general intelligence (AGI)’ in them^8^, others see them as ‘stochastic parrots’—that is, systems that only regurgitate what they have been trained on^9^ and that show inherent limitations owing to the way they are trained^10^. Nevertheless, the promise of these models is that they have shown the ability to solve a wide variety of tasks they have not been explicitly trained on^11–13^.

Chemists and materials scientists have quickly caught on to the mounting attention given to LLMs, with some voices even suggesting that ‘the future of chemistry is language’^14^. This statement is motivated by a growing number of reports that use LLMs to predict properties of molecules or materials^2,15–19^, optimize reactions^20,21^, generate materials^22–25^, extract information^26–33^ or even to prototype systems that can autonomously perform experiments in the physical world based on commands provided in natural language^5–7^.

In addition, since a lot—if not most—of the information about chemistry is currently stored and communicated in text, there is a strong reason to believe that there is still a lot of untapped potential in LLMs for chemistry and materials science^34^. For instance, most insights in chemical research do not directly originate from data stored in databases but rather from the scientists interpreting the data. Many of these insights are in the form of text in scientific publications. Thus, operating on such texts might be our best way of unlocking these insights and learning from them. This might ultimately lead to general copilot systems for chemists that can provide answers to questions or even suggest new experiments on the basis of vastly more information than a human could ever read.

However, the rapid increase in capabilities of chemical ML models led (even before the recent interest in LLMs) to concerns about the potential for the dual use of these technologies, for example, for the design of chemical weapons^35–40^. To some extent, this is not surprising as any technology that, for instance, is used to design non-toxic molecules can also be used inversely to predict toxic ones (even though the synthesis would still require access to controlled physical resources and facilities). Still, it is essential to realize that the user base of LLMs is broader than that of chemistry and materials science experts who can critically reflect on every output these models produce. For example, many students frequently consult these tools—perhaps even to prepare chemical experiments^41^. This also applies to users from the general public, who might consider using LLMs to answer questions about the safety of chemicals. Thus, for some users, misleading information—especially about safety-related aspects—might lead to harmful outcomes. However, even for experts, chemical knowledge and reasoning capabilities are essential as they will determine the capabilities and limitations of their models in their work, for example, in copilot systems for chemists. Unfortunately, apart from exploratory reports, such as by prompting leading models with various scientific questions^13^, there is little systematic evidence on how LLMs perform compared with expert (human) chemists.

Thus, to better understand what LLMs can do for the chemical sciences and where they might be improved with further developments, evaluation frameworks are needed to allow us to measure progress and mitigate potential harms systematically. For the development of LLMs, evaluation is currently primarily performed via standardized benchmark suites such as BigBench^42^ or the LM Eval Harness^43^. Among 204 tasks (such as linguistic puzzles), the former contains only 2 tasks classified as ‘chemistry related’, whereas the latter contains no specific chemistry tasks. Owing to the lack of widely accepted standard benchmarks, the developers of chemical language models^16,44–47^ frequently utilize language-interfaced^48^ tabular datasets such as the ones reported in MoleculeNet^49,50^, Therapeutic Data Commons^51^, safety databases^52^ or MatBench^53^. In these cases, the models are evaluated on predicting very specific properties of molecules (for example, solubility, toxicity, melting temperature or reactivity) or on predicting the outcome of specific chemical reactions. This, however, only gives a very limited view of the general chemical capabilities of the models.

While some benchmarks based on university entrance exams^54,55^ or automatic text mining^56–58^ have been proposed, none of them have been widely accepted. This is probably because they cannot automatically be used with black box (or tool-augmented) systems, do not cover a wide range of topics and skills or are not carefully validated by experts. On top of that, the existing benchmarks are not designed to be used with models that support special treatment of molecules or equations and do not provide insights on how the models compare relative to experts^49^.

**Fig. 1:**
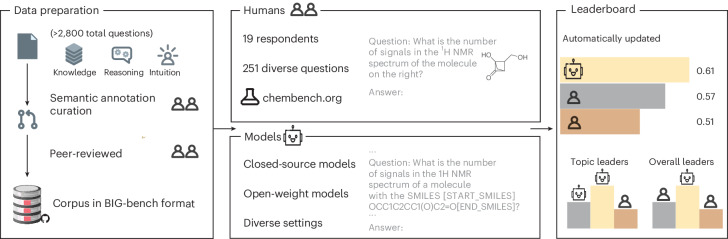
Overview of the ChemBench framework. The different components of the ChemBench framework. The framework’s foundation is the benchmark corpus comprising thousands of questions and answers that we manually or semi-automatically compiled from various sources in a format based in the one introduced in the BIG-bench benchmark (Extended Data Fig. 1). Questions are classified on the basis of topics, required skills (reasoning, calculation, knowledge and intuition) and difficulty levels. We then used this corpus to evaluate the performance of various models and tool-augmented systems using a custom framework. To provide a baseline, we built a web application that we used to survey experts in chemistry. The results of the evaluations are then compiled in publicly accessible leaderboards (Supplementary Note 15), which we propose as a foundation for evaluating future models.

In this work, we report a benchmarking framework (Fig. 1), which we call ChemBench, and use it to reveal the limitations of current frontier models for use in the chemical sciences. Our benchmark consists of 2,788 question–answer pairs compiled from diverse sources (1,039 manually generated and 1,749 semi-automatically generated). Our corpus measures reasoning, knowledge and intuition across a large fraction of the topics taught in undergraduate and graduate chemistry curricula. It can be used to evaluate any system that can return text (that is, including tool-augmented systems).

To contextualize the scores, we also surveyed 19 experts in chemistry on a subset of the benchmark corpus to be able to compare the performance of current frontier models with (human) chemists of different specializations. In parts of the survey, the volunteers were also allowed to use tools, such as web search, to create a realistic setting.

## Results and discussion

### Benchmark corpus

**Fig. 2:**
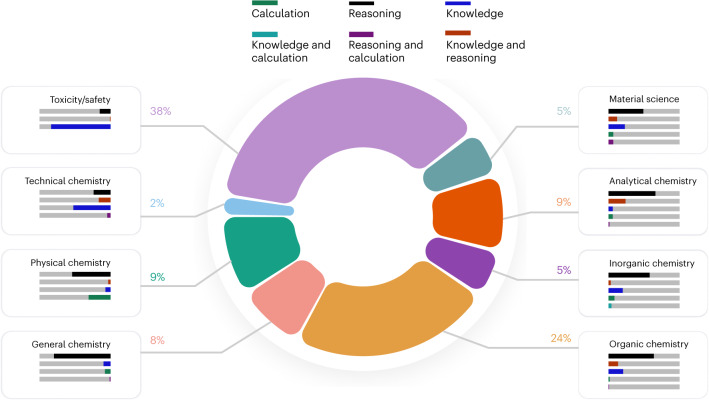
Distribution of topics and required skills. The distribution of questions across various chemistry topics, along with the primary skills required to address them. The topics were manually classified, showing a varied representation across different aspects of chemistry. Each topic is associated with a combination of three key skills: calculation, reasoning and knowledge, as indicated by the coloured bars. ChemBench samples encompass diverse topics and diverse skills, setting a high bar for LLMs to demonstrate human-competitive performance across a wide range of chemistry tasks.

To compile our benchmark corpus, we utilized a broad list of sources (Methods), ranging from completely novel, manually crafted questions over university exams to semi-automatically generated questions based on curated subsets of data in chemical databases. For quality assurance, all questions have been reviewed by at least two scientists in addition to the original curator and automated checks. Importantly, our large pool of questions encompasses a wide range of topics and question types (Fig. 2). The topics range from general chemistry to more specialized fields such as inorganic, analytical or technical chemistry. We also classify the questions on the basis of what skills are required to answer them. Here, we distinguish between questions that require knowledge, reasoning, calculation, intuition or a combination of these. Moreover, the annotator also classifies the questions by difficulty to allow for a more nuanced evaluation of the models’ capabilities.

While many existing benchmarks are designed around multiple-choice questions (MCQ), this does not reflect the reality of chemistry education and research. For this reason, ChemBench samples both MCQ and open-ended questions (2,544 MCQ and 244 open-ended questions). In addition, ChemBench samples different skills on various difficulty levels: from basic knowledge questions (as knowledge underpins reasoning processes^59,60^) to complex reasoning tasks (such as finding out which ions are in a sample given a description of observations). We also include questions about chemical intuition, as demonstrating human-aligned preferences is relevant for applications, such as hypothesis generation or optimization tasks^61^.

#### ChemBench-Mini

It is important to note that a smaller subset of the corpus might be more practical for routine evaluations^62^. For instance, Liang et al.^63^ report costs of more than US$10,000 for application programming interface (API) calls for a single evaluation on the widely used Holistic Evaluation of Language Models benchmark. To address this, we also provide a subset (ChemBench-Mini, 236 questions) of the corpus that was curated to be a diverse and representative subset of the full corpus. While it is impossible to comprehensively represent the full corpus in a subset, we aimed to include a maximally diverse set of questions and a more balanced distribution of topics and skills (see Methods for details on the curation process). Our human volunteers answered all the questions in this subset.

### Model evaluation

#### Benchmark suite design

Because the text used in scientific settings differs from typical natural language, many models have been developed that deal with such text in a particular way. For instance, the Galactica model^64^ uses special encoding procedures for molecules and equations. Current benchmarking suites, however, do not account for such special treatment of scientific information. To address this, ChemBench encodes the semantic meaning of various parts (for example, chemicals, units or equations) of the question or answer. For instance, molecules represented in simplified molecular input line-entry system (SMILES) are enclosed in [START_SMILES][\END_SMILES] tags. This allows the model to treat the SMILES string differently from other text. ChemBench can seamlessly handle such special treatment in an easily extensible way because the questions are stored in an annotated format.

Since many widely utilized LLM systems only provide access to text completions (and not the raw model outputs), ChemBench is designed to operate on text completions. This is also important given the growing number of tool-augmented systems that are deemed essential for building chemical copilot systems. Such systems can augment the capabilities of LLMs through the use of external tools such as search APIs or code executors^65–67^. In those cases, the LLM which returns the probabilities for various tokens (that is, text fragments) represents only one component and it is not clear how to interpret those probabilities in the context of the entire system. The text completions, however, are the system’s final outputs, which would also be used in a real-world application. Hence, we use them for our evaluations^68^.

#### Overall system performance

**Fig. 3:**
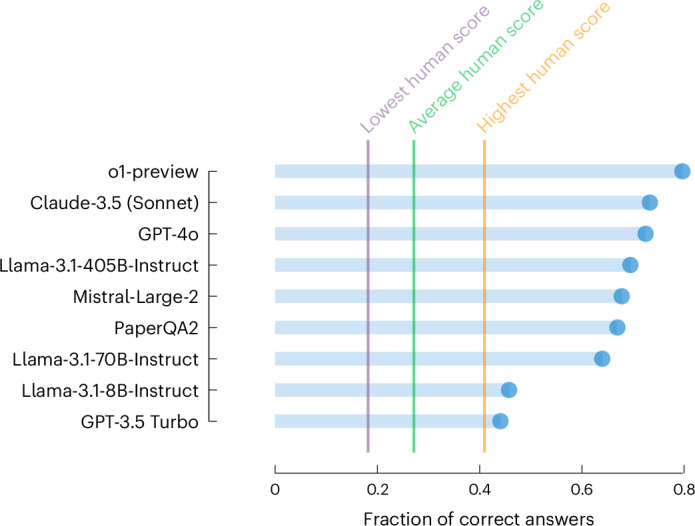
Performance of models and humans on ChemBench-Mini. The percentage of questions that the models answered correctly. Horizontal bars indicate the performance of various models and highlight statistics of human performance. The evaluation we use here is very strict as it only considers a question answered correctly or incorrectly, partially correct answers are also considered incorrect. Supplementary Fig. 3 provides an overview of the performance of various models on the entire corpus. PaperQA2 (ref. ^33^) is an agentic system that can also search the literature to obtain an answer. We find that the best models outperform all humans in our study when averaged over all questions (even though humans had access to tools, such as web search and ChemDraw, for a subset of the questions).

To understand the current capabilities of LLMs in the chemical sciences, we evaluated a wide range of leading models^69^ on the ChemBench corpus, including systems augmented with external tools. An overview of the results of this evaluation is presented in Fig. 3 (all results can be found in Supplementary Fig. 4 and Supplementary Table 5). In Fig. 3, we show the percentage of questions that the models answered correctly. Moreover, we show the worst, best and average performance of the experts in our study, which we obtained via a custom web application (chembench.org) that we used to survey the experts. Remarkably, the figure shows that the leading LLM, o1-preview, outperforms the best human in our study in this overall metric by almost a factor of two. Many other models also outperform the average human performance. Interestingly, Llama-3.1-405B-Instruct shows performance that is close to the leading proprietary models, indicating that new open-source models can also be competitive with the best proprietary models in chemical settings.

Notably, we find that models are still limited in their ability to answer knowledge-intensive questions (Supplementary Table 5); that is, they did not memorize the relevant facts. Our results indicate that this is not a limitation that could be overcome by simple application of retrieval augmented generation systems such as PaperQA2. This is probably because the required knowledge cannot easily be accessed via papers (which is the only type of external knowledge PaperQA2 has access to) but rather by lookup in specialized databases (for example, PubChem and Gestis), which the humans in our study also used to answer such questions (Supplementary Fig. 17). This indicates that there is still room for improving chemical LLMs by training them on more specialized data sources or integrating them with specialized databases.

In addition, our analysis shows that the performance of models is correlated with their size (Supplementary Fig. 11). This is in line with observations in other domains, but also indicates that chemical LLMs could, to some extent, be further improved by scaling them up.

#### Performance per topic

**Fig. 4:**
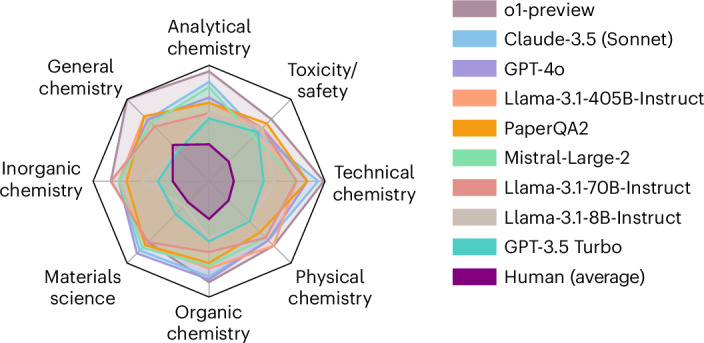
Performance of the models and humans on the different topics on ChemBench-Mini. The radar plot shows the performance of the models and humans on the different topics of ChemBench-Mini. Performance is measured as the fraction of questions that were answered correctly by the models. The best score for every dimension is 1 (all questions answered correctly) and the worst is 0 (no question answered correctly). A larger coloured area indicates a better performance. This figure shows the performance on ChemBench-Mini. The performance of models on the entire corpus is presented in Supplementary Fig. 3.

To obtain a more detailed understanding of the performance of the models, we also analysed the performance of the models in different subfields of the chemical sciences. For this analysis, we defined a set of topics (Methods) and classified all questions in the ChemBench corpus into these topics. We then computed the percentage of questions that the models or experts answered correctly for each topic and present them in Fig. 4. In this spider chart, the worst score for every dimension is zero (no question answered correctly) and the best score is one (all questions answered correctly). Thus, a larger coloured area indicates a better performance.

One can observe that this performance varies across models and topics. While general and technical chemistry receive relatively high scores for many models, this is not the case for topics such as toxicity and safety or analytical chemistry.

In the subfield of analytical chemistry, the prediction of the number of signals observable in a nuclear magnetic resonance spectrum proved difficult even for the best models (for example, 22% correct answers for o1-preview). Importantly, while the human experts are given a drawing of the compounds, the models are only shown the SMILES string of a compound and have to use this to reason about the symmetry of the compound (that is, to identify the number of diasterotopically distinct protons, which requires reasoning about the topology and structure of a molecule).

These findings also shine an interesting light on the value of textbook-inspired questions. A subset of the questions in ChemBench are based on textbooks targeted at undergraduate students. On those questions, the models tend to perform better than on some of our semi-automatically constructed tasks (Supplementary Fig. 5). For instance, while the overall performance in the chemical safety topic is low, the models would pass the certification exam according to the German Chemical Prohibition Ordinance on the basis of a subset of questions we sampled from the corresponding question bank (for example, 71% correct answers for GPT-4, 61% for Claude-3.5 (Sonnet) and 3% for the human experts). While those findings are impacted by the subset of questions we sampled, the results still highlight that good performance on such question bank or textbook questions does not necessarily translate to good performance on other questions that require more reasoning or are further away from the training corpus^10^. The findings also underline that such exams might have been a good surrogate for the general performance of skills for humans, but their applicability in the face of systems that can consume vast amounts of data is up for debate.

We also gain insight into the models’ struggles with chemical reasoning tasks by examining their performance as a function of molecular descriptors. If the model would answer questions after reasoning about the structures, one would expect the performance to depend on the complexity of the molecules. However, we find that the models’ performance does not correlate with complexity indicators (Supplementary Note 5). This indicates that the models may not be able to reason about the structures of the molecules (in the way one might expect) but instead rely on the proximity of the molecules to the training data^10^.

It is important to note that the model performance for some topics, however, is slightly underestimated in the current evaluation. This is because models provided via APIs typically have safety mechanisms that prevent them from providing answers that the provider deems unsafe. For instance, models might refuse to provide answers about cyanides. Statistics on the frequency of such refusals are presented in Supplementary Table 8. To overcome this, direct access to the model weights would be required, and we strive to collaborate with the developers of frontier models to overcome this limitation in the future. This is facilitated by the tooling ChemBench provides, thanks to which contributors can automatically add new models in an open science fashion.

#### Judging chemical preference

One interesting finding of recent research is that foundation models can judge interestingness or human preferences in some domains^61,70^. If models could do so for chemical compounds, this would open opportunities for novel optimization approaches. Such open-ended tasks, however, depend on an external observer defining what interestingness is^71^. Here, we posed models the same question that Choung et al.^72^ asked chemists at a drug company: ‘which of the two compounds do you prefer?’ (in the context of an early virtual screening campaign setting; see Supplementary Table 2 for an example). Despite chemists demonstrating a reasonable level of inter-rater agreement, our models largely fail to align with expert chemists’ preferences. Their performance is often indistinguishable from random guessing, even though these same models excel in other tasks in ChemBench (Supplementary Table 5). This indicates that using preference tuning for chemical settings could be a promising approach to explore in future research.

#### Confidence estimates

One might wonder whether the models can estimate if they can answer a question correctly. If they could do so, incorrect answers would be less problematic.

To investigate this, we prompted^68^ some of the top-performing models to estimate, on an ordinal scale, their confidence in their ability to answer the question correctly (see Methods for details on the methodology and comparison to logit-based approaches).

**Fig. 5:**
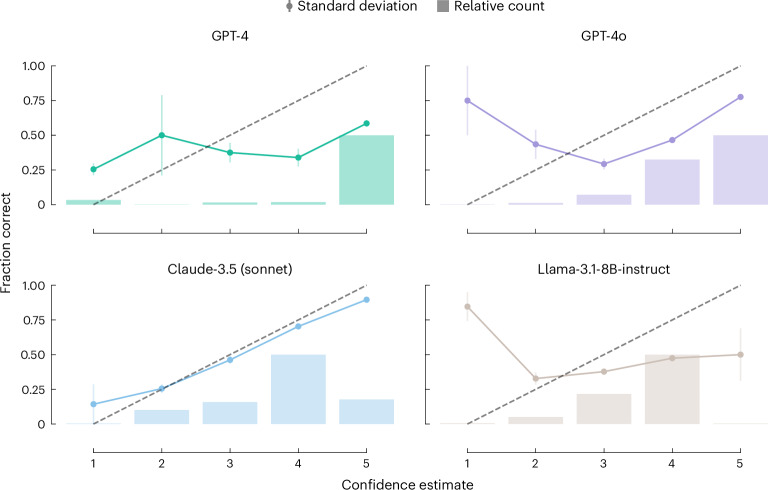
Reliability and distribution of confidence estimates. For this analysis, we used verbalized confidence estimates from the model. The models were prompted to return a confidence score on an ordinal scale to obtain those estimates. The line plot shows the average fraction of correctly answered questions for each confidence level. The bar plot shows the distribution of confidence estimates. The error bars indicate the standard deviation for each confidence level (for which the number of samples is given by the height of the bar). A confidence estimate would be well calibrated if the average fraction of correctly answered questions increases with the confidence level. The dashed black line indicates this ideal behaviour, which would be monotonically increasing correctness with higher levels of confidence. We use colours to distinguish the different models, as indicated in the titles of the subplots. We find that most models are not well calibrated and provide misleading confidence estimates.

In Fig. 5, we show that for some models, there is no meaningful correlation between the estimated difficulty and whether the models answered the question correctly or not. For applications in which humans might rely on the models to provide answers with trustworthy uncertainty estimates, this is a concerning observation highlighting the need for critical reasoning in the interpretation of the model’s outputs^34,73^. For example, for the questions about the safety profile of compounds, GPT-4 reported a confidence of 1.0 (on a scale of 1–5) for the one question it answered correctly and 4.0 for the six questions it answered incorrectly. While, on average, the verbalized confidence estimates from Claude-3.5 (Sonnet) seem better calibrated (Fig. 5), they are still misleading in some cases. For example, for the questions about the labelling of chemicals (GHS) pictograms Claude-3.5 (Sonnet) returns an average score of 2.0 for correct answers and 1.83 for incorrect answers.

## Conclusions

On the one hand, our findings underline the impressive capabilities of LLMs in the chemical sciences: leading models outperform domain experts in specific chemistry questions on many topics. On the other hand, there are still striking limitations. For very relevant topics, the answers that models provide are wrong. On top of that, many models are not able to reliably estimate their own limitations. Yet, the success of the models in our evaluations perhaps also reveals more about the limitations of the questions we use to evaluate models—and chemists—than about the models themselves. For instance, while models perform well on many textbook questions, they struggle with questions requiring more reasoning about chemical structures (for example, number of isomers or nuclear magnetic resonance peaks). Given that the models outperformed the average human in our study, we need to rethink how we teach and examine chemistry. Critical reasoning is increasingly essential, and rote solving of problems or memorization of facts is a domain in which LLMs will continue to outperform humans (when trained on the right training corpus).

Our findings also highlight the nuanced trade-off between breadth and depth of evaluation frameworks. The analysis of model performance on different topics shows that models’ performance varies widely across the subfields they are tested on. However, even within a topic, the performance of models can vary widely depending on the type of question and the reasoning required to answer it.

The current evaluation frameworks for chemical LLMs are primarily designed to measure the performance of the models on specific property prediction tasks. They cannot be used to evaluate reasoning or systems built for scientific applications. Thus, we had little understanding of the capabilities of LLMs in the chemical sciences. Our work shows that carefully curated benchmarks can provide a more nuanced understanding of the capabilities of LLMs in the chemical sciences. Importantly, our findings also illustrate that more focus is required in developing better human–model interaction frameworks, given that models cannot estimate their limitations.

Although our findings indicate many areas for further improvement of LLM-based systems, such as agents (more discussion in Supplementary Note 11), it is also important to realize that clearly defined metrics have been the key to the progress of many fields of ML, such as computer vision. Although current systems might be far from reasoning like a chemist, our ChemBench framework will be a stepping stone for developing systems that come closer to this goal.

## Methods

### Curation workflow

**Extended Data Fig. 1:**
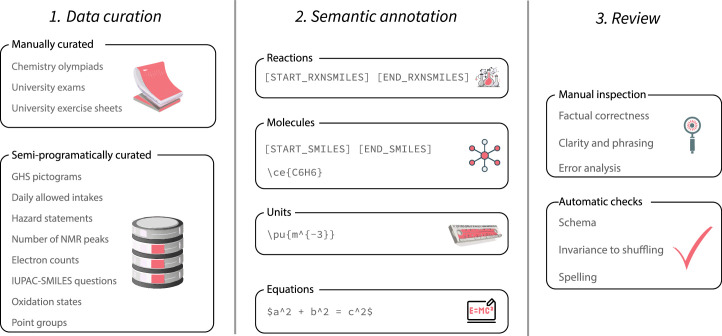
Overview of the workflow for the assembly of the ChemBench Corpus. To assemble the ChemBench corpus, we first collected questions from various sources. Some tasks were manually curated, others semi-programmatically. We added semantic annotations for all questions to make them compatible with systems that use special processing for modalities that are not conventional natural text. We reviewed the questions using manual and automatic methods before adding them to the corpus.

For our dataset, we curated questions from existing exams or exercise sheets but also programmatically created new questions (see Supplementary Table 3 for more details). Questions were added via Pull Requests on our GitHub repository and only merged into the corpus after passing manual review (Extended Data Fig. 1) as well as automated checks (for example, for compliance with a standardized schema).

To ensure that the questions do not enter a training dataset, we use the same canary string as the BigBench project. This requires that LLM developers filter their training dataset for this canary string^4,42^.

#### Manually curated questions

Manually curated questions were sourced from various sources, including university exams, exercises and question banks. Extended Data Table 1 presents an overview of the sources of the manually curated questions.

#### Semi-programmatically generated questions

In addition to the manually curated questions, we also generated questions programmatically. An overview of the sources of the semi-programmatically generated questions is provided in Supplementary Table 3.

#### Chemical preference data

These questions assess the ability to establish a ‘preference’, such as favouring a specific molecule. Chemical preference is of major importance in drug discovery projects, where the optimization process to reach the desired molecular properties is a process that takes several years within a chemist’s career. Our data corpus is adapted from the published dataset by Choung et al.^72^, which consists of more than 5,000 question–answer pairs about chemical intuition. To build the dataset, they presented 35 medicinal chemists with two different molecules, asking them what molecule they would like to continue with when imaging an early virtual screening campaign setting. The question was designed so the scientists do not spend much time answering it, relying only on their feelings or ‘chemical preference’.

To understand whether the capabilities of the leading models align with the preferences of professional chemists, we randomly selected 1,000 data points from the original dataset to create a meaningful evaluation set, where molecules are represented as SMILES. To ablate the effect of different molecular representations, we only considered questions for which we could obtain International Union of Pure and Applied Chemistry names for both the molecules present.

### Model evaluation workflow

A graphical overview of the pipeline is presented in Supplementary Fig. 12.

#### Prompting

We employ distinct prompt templates tailored for completion and instruction-tuned models to maintain consistency with the training. As explained later, we impose constraints on the models within these templates to receive responses in a specific format so that robust, fair and consistent parsing can be performed. Certain models are trained with special annotations and LaTeX syntax for scientific notations, chemical reactions or symbols embedded within the text. For example, all the SMILES representations are encapsulated within [START_SMILES][\END_SMILES] in Galactica^64^. Our prompting strategy consistently adheres to these details in a model-specific manner by post-processing LaTeX syntax, chemical symbols, chemical equations and physical units (by either adding or removing wrappers). This step can be easily customized in our codebase, and we provide presets for the models we evaluated.

#### Parsing

Our parsing workflow is multistep and primarily based on regular expressions. In the case of instruction-tuned models, we first identify the [ANSWER][\ANSWER] environment that we prompt the model to report the answer in. In the case of completion models, this step is skipped. From there, we attempt to extract the relevant enumeration letters (for MCQ) or numbers. In the case of numbers, our regular expression was engineered to deal with various forms of scientific notation. As initial tests indicated that models sometimes return integers in the form of words, for example, ‘one’ instead of ‘1’, we also implemented a word-to-number conversion using regular expressions. If these hard-coded parsing steps fail, we use a LLM, for example, Claude-3.5 (Sonnet), to parse the completion (Supplementary Note 8 provides more details on this step).

#### Models

For all models, we performed inference using greedy decoding (that is, temperature 0). We used the API endpoints provided by the model developers and those provided by Groq. PaperQA2 was used (in August 2024) via an API provided by FutureHouse.

### Confidence estimate

To estimate the models’ confidence, we prompted them with the question (and answer options for MCQ) and the task to rate their confidence to produce the correct answer on a scale from 1 to 5. We decided to use verbalized confidence estimates^68^ since we found those to be closer to current practical use cases than other prompting strategies, which might be more suitable when implemented in systems. In addition, this approach captures semantic uncertainty, which is not the same as the probability of a token being given a sequence of tokens (that is, the uncertainty one obtains from logit-based approaches). On top of that, many proprietary models do not provide access to the logits, making this approach more general. In Supplementary Note 12, we provide more details and comparisons with a logit-based approach.

### Human baseline

#### Question selection

Several design choices were made when selecting ChemBench-Mini. Firstly, from the full dataset, we kept all the questions labelled as advanced. In this way, we can obtain a deeper insight into the capabilities of LLMs on advanced tasks when compared with actual chemists. Secondly, we sample a maximum of three questions across all possible combinations of categories (that is, knowledge or reasoning) and topics (for example, organic chemistry and physical chemistry). Thirdly, we do not include any intuition questions in this subset because the intended use of ChemBench-Mini is to provide a fast and fair evaluation of LLMs independent of any human baseline. In total, 236 questions have been sampled for ChemBench-Mini. Then, this set is divided into two subsets on the basis of the aforementioned combinations. One of the question subsets allows tool use, and the other does not.

#### Study design

Human volunteers were asked the questions in a custom-built web interface (Supplementary Note 10), which rendered chemicals and equations. Questions were shown in random order, and volunteers were not allowed to skip questions. For a subset of the questions, the volunteers were allowed to use external tools (excluding other LLM or asking other people) to answer the questions. Before answering questions, volunteers were asked to provide information about their education and experience in chemistry. The study was conducted in English.

#### Human volunteers

Users were open to reporting about their experience in chemistry. Overall, 16 did so. Out of those, 2 are beyond a first postdoc, 13 have a master’s degree (and are currently enroled in Ph.D. studies) and 1 has a bachelor’s degree. For the analysis, we excluded volunteers with less than 2 years of experience in chemistry after their first university-level course in chemistry.

#### Comparison with models

For the analysis, we treated each human as a model. We computed the topic aggregated averages per human for analyses grouped by topic and then averaged over all humans. The performance metrics reported for models in the main text are computed on the same questions that the humans answered. Metrics for the entire corpus are reported in Supplementary Note 4.

### Data annotation

In the curation of our dataset, we manually assigned difficulty levels and required skills to each question. We used the following guidelines for these annotations: calculation is required if answering a question would require the use of a calculator, knowledge is required if answering a question requires non-trivial knowledge of facts (for example, the H/P statements of chemicals). Reasoning is required if answering a question requires multiple reasoning steps. Basic questions only require those skills up to the high school level. Advanced questions would require an expert multiple minutes or hours to answer.

### Inclusion and ethics statement

The authors confirm that they have complied with all relevant ethical regulations, according to the Ethics Commission of the Friedrich Schiller University Jena (which decided that the study is ethically safe). Informed consent was obtained from all volunteers.

### Reporting summary

Further information on research design is available in the Nature Portfolio Reporting Summary linked to this article.

## Online content

Any methods, additional references, Nature Portfolio reporting summaries, source data, extended data, supplementary information, acknowledgements, peer review information; details of author contributions and competing interests; and statements of data and code availability are available at 10.1038/s41557-025-01815-x.

## Supplementary information

Supplementary Information Supplementary Notes 1–15 and Figs. 1–19.

Reporting Summary

## Data Availability

The data for ChemBench is available via GitHub at https://github.com/lamalab-org/chembench and via Zenodo at https://zenodo.org/records/14010212 (ref. ^74^).

## Extended data

**Extended Data Table 1:** Overview of sources of the curated questions

Source	Count
Semi-automatically generated	1749
URL	375
Textbook	206
Exam	149
IChO	149
No source	139
Lectures	21

The table provides an overview of the types of sources the questions have been curated from. Detailed sources are available in the source data on GitHub. Questions without a source have been curated completely from scratch. Questions based on lecture notes or URLs have been curated based on content presented in those resources. All questions have been rephrased, annotated, and reviewed before being added to the corpus.

## Code availability

The code for ChemBench is available via GitHub at https://github.com/lamalab-org/chembench and via Zenodo at https://zenodo.org/records/14010212 (ref. ^74^) The code for the app for our human baseline study is available via GiHub at https://github.com/lamalab-org/chem-bench-app.
