# Supplementary material for: A framework for evaluating the chemical knowledge and reasoning abilities of large language models against the expertise of chemists
Source: Nat Chem. 2025 May 20;17(7):1027–34. doi: 10.1038/s41557-025-01815-x (PMC12226332; doi:10.1038/s41557-025-01815-x)
Supplement: Supplementary file 1 — Supplementary Notes 1–15 and Figs. 1–19. [file 41557_2025_1815_MOESM1_ESM.pdf]

nature chemistry

Supplementary information https://doi.org/10.1038/s41557-025-01815-x

A framework for evaluating the chemical knowledge and reasoning abilities of large language models against the expertise of chemists

In the format provided by the authors and unedited

Contents

Supplementary Note 1 Desired properties of a chemistry benchmark 2

Supplementary Note 2 Related work 3

Supplementary Note 3 Benchmark corpus 4

Supplementary Note 3.1 Curation workflow 4

Supplementary Note 3.2 Composition 5

Supplementary Note 3.3 Sources of semi-programatically generated questions 11

Supplementary Note 4 Model performance 13

Supplementary Note 5 Performance as a function of molecular features 22

Supplementary Note 6 Influence of model scale 24

Supplementary Note 7 Refusal detection 25

Supplementary Note 8 LLM Parsing 25

Supplementary Note 9 Implementation 27

Supplementary Note 10 Human baseline 29

Supplementary Note 11 Tool augmented models 33

Supplementary Note 12 Confidence estimates 34

Supplementary Note 13 Impact of sampling temperature 36

Supplementary Note 14 Summary of trends and recommendations 37

Supplementary Note 15 Leaderboard 38

Supplementary Information

Supplementary Note 1 Desired properties of a chemistry benchmark

- *End-to-end automation.* For model development, the evaluations must be run many times (e.g., on regular intervals of a training run). Approaches that rely on humans scoring the answers of a system[1–3] can thus not be used.
- *Careful validation by experts.* Manual curation is needed to minimize the number of incorrect or unanswerable questions.[4] This is motivated by the observation that many widely used benchmarks are plagued by noisiness.[5,6]
- *Usable with models that support special treatment of molecules.* Some models, such as Galactica[7], use special tokenization or encoding procedures for molecules or equations. The benchmark system must encode the semantic meaning of various parts of the question or answer to support this.
- *Usable with black box systems.* Many relevant systems do not provide access to model weights or raw logits. This might be the case because the systems are proprietary or because they involve not only large language models (LLMs) but also external tools such as search application programming interfaces (APIs) or code executors.[8–10] Thus, a benchmark should not assume access to the raw model outputs but be able to operate on text completions.
- *Probing capabilities beyond answering of multiple-choice questions (MCQs).* In real-world chemistry, as well as higher-level university education, multiple-choice questions are seldom utilized. Yet, most benchmarking frameworks focus on the MCQ setting because of the ease of evaluation. Realistic evaluations must measure capabilities beyond answering MCQ.
- *Cover a diverse set of topics.* Chemistry, as the "central science", bridges multiple disciplines.[11] To even just approximate "chemistry capabilities", the topics covered by a chemistry benchmark must be very diverse.
- *Cover diverse skills.* To holistically judge performance, it is important to cover questions that rely on reasoning, calculation, knowledge, and intuition.
- *Cover a range of difficulty levels.* To allow for a continuous measure of improvement for a range of different (evolving) systems, a benchmark should cover a wide range of difficulty levels.

- *Impossible to completely solve with current models.* A benchmark should contain questions that are impossible to solve with current models. The benchmark provides no useful signal if current models can solve all questions.

Supplementary Note 2 Related work

Existing benchmarks such as those from Guo et al.[12], Sun et al.[13], Schulze Balhorn et al.[1], Cai et al.[14], Rein et al.[15] fail to comply with most of the requirements stipulated above. While these benchmarks could provide valuable insights in the short term, they cannot follow the rapid additions to the LLM space. ChemBench aims to correct this through a set of developments: compatibility with BigBench, end-to-end automation, a particular focus on chemical safety, employment of diverse prompting strategies, and specialized notation for molecules and mathematical symbols. Moreover, our robust framework, including the platform `chembench.org`, will engage the community in open-source contributions.

Supplementary Note 3 Benchmark corpus

To ensure maximal interoperability with existing benchmarks or tools, we curated the data in an extended form of the widely used BigBench format.[16] This also implies that future baselines can be built on top of our infrastructure if saved in the same format.

Supplementary Note 3.1 Curation workflow

Questions were added via pull requests to the ChemBench repository on GitHub. This allowed for a manual review of each question by expert reviewers (with backgrounds in chemistry, materials science, chemical engineering, and physics). The reviews were conducted directly on the GitHub platform, where our entire curation history is also publicly available.

The general guidelines followed by the reviewer are the following:

- *Originality:* Questions should not be readily findable online or in other easily accessible sources (example `https://github.com/lamalab-org/chem-bench/pull/392#discussion_r1694299474`)
- *Ambiguity:* Questions with unclear wording or multiple interpretations (example `https://github.com/lamalab-org/chem-bench/pull/420#discussion_r1698147159`)
- *Factual or heuristic Errors:* Questions containing factual inaccuracies or misconceptions are not included (example `https://github.com/lamalab-org/chem-bench/pull/389#discussion_r1686187301`)
- *Clarity and Difficulty:* They should pose a challenge and encourage exploration within the chemical domain (example `https://github.com/lamalab-org/chem-bench/pull/391#discussion_r1679276714`)
- *Out of Scope:* Questions outside the realm of chemistry are rejected.
- *Contribution to Dataset Diversity:* Questions should cover a wide range of chemical concepts and sub-disciplines. They should add value by expanding the breadth of the dataset. That is, questions already multiple (>10) times in the corpus in a similar form are rejected.

Reviewers also solved the questions to verify the answers. They also performed web searches to ensure questions were not easily found online. The reviewers often guided the revision process to ensure the question aligned with the guidelines. Questions that don't meet the criteria are either rejected or suggested for revision, and

most often, they are modified to a new question. Reviewers also provide feedback on the skill and difficulty annotations.

In addition to the manual review, we also performed automated checks to ensure the quality of the questions. The schemas, LaTeX templating, and other formatting aspects are checked automatically using GitHub Actions.

While adding questions from existing benchmarks might seem to be another good source of semi-automatically generated data, we prioritized the diversity of the data and avoided data contamination while addressing the guidelines above and in Supplementary Note 1. However, even though we decided not to include questions from other previously published chemistry-focused benchmarks into the ChemBench corpus, the framework is flexible enough to be readily extended with questions from other benchmarks.

Supplementary Note 3.2 Composition

Supplementary Fig. 1 shows the distribution of topics and required skills in the human subset of the ChemBench corpus.

The corpus of the questions in ChemBench, as shown in Supplementary Table 1, can be divided according to which chemical topic they belong.

Supplementary Table 1 | Examples for each of the topics considered in the evaluation of the ChemBench corpus. The table shows the percentage of questions in the corpus that belong to each topic, as well as example questions.

**Analytical Chemistry** 148 Questions (38.51%)
Which of the following analytical methods is most appropriate for performing a survey analysis of a solid sample containing various metals?<br>A. X-ray fluorescence analysis<br>B. Differential pulse polarography<br>C. Flame-atomic absorption spectroscopy<br>D. Gas chromatography with flame ionization detector<br>E. Hydride generation atomic absorption spectroscopy
**Chemical Preference** 1001 Questions (51.15%)
Imagine an early virtual screening campaign setting (accounting for simple aspects such as oral availability and small molecular profile, but no other modalities such as covalency or bifunctionality). Which of the following two candidates would you prefer for further development?<br>A. [START_SMILES]N#Cc1ccc(OCCCN2CC3CN-(CCNS(=O)(=O)c4ccc(F)cc4)CC(C2)O3)cc1[END_SMILES]

B. [START_SMILES]O=C1CC(c2ccc(CC(NS(=O)(=O)-c3cc(Cl)cc(Cl)c3)c3nc4ccccc4[nH]3)cc2)S(=O)(=O)N1[END_SMILES]

General Chemistry 150 Questions (50.67%)

Which of the following salts is an acidic salt?
A. NH_4Cl
B. Na_2CO_3
C. NaH_2PO_4
D. Zn(OH)Cl

Inorganic Chemistry 101 Questions (56.44%)

What is the oxidation number of the metal in the compound $[ZrF_7]_3^-$?

Materials Science 10 Questions (30.00%)

For NMR analysis, you need to digest the MOF in a strong acid to remove the linker and leave the metal clusters intact. Why would one choose HF over HCl for this purpose?
A. F^- forms a stable bonds to the metal ions
B. HF has a better water solubility than HCl
C. HF has a higher boiling point than HCl
D. HF is a weaker acid than HCl

Organic Chemistry 334 Questions (59.58%)

What is the reaction mechanism that describes the following reaction (represented using reaction SMILES) [START_RXNSMILES]CCCl.CO[Na]>>[Na]Cl.CCOC[END_RXNSMILES]?
A. E_1
B. E_{cb}
C. S_N1
D. S_N2

Physical Chemistry 142 Questions (53.52%)

The Born-Oppenheimer (BO) approximation is widely used in computational chemistry, but its accuracy can vary depending on the system. Among the following options, for which system is the Born-Oppenheimer approximation likely to be least applicable?
A. C_{60}
B. CH_4
C. $Fe(CO)_5$
D. H_2^+
E. NaCl

Technical Chemistry 40 Questions (47.50%)

Which of the following statements is true about the different types of ideal reactors?
A. In a batch reactor, the composition is uniformly mixed and remains the same throughout the reactor and at the exit
B. In a batch reactor, the fluid passes through the reactor with no mixing of earlier and later entering fluid
C. In a mixed flow reactor, the composition changes with time but is uniform everywhere within the reactor
D. In a plug flow reactor, the fluid moves in single flow through the reactor with no mixing and no overtaking

Toxicity/Safety 675 Questions (33.19%)

Pindolol and propranolol are (relatively nonselective) antagonists at β_1- and β_2-adrenoceptors. However, pindolol is a partial agonist, whereas propranolol is a pure antagonist. What follows from this?
A. Pindolol has a greater therapeutic range than propranolol
B. Pindolol has a longer half-life than propranolol
C. Pindolol has intrinsic activity
D. Pindolol is more lipophilic than propranolol
E. Pindolol is more potent than propranolol

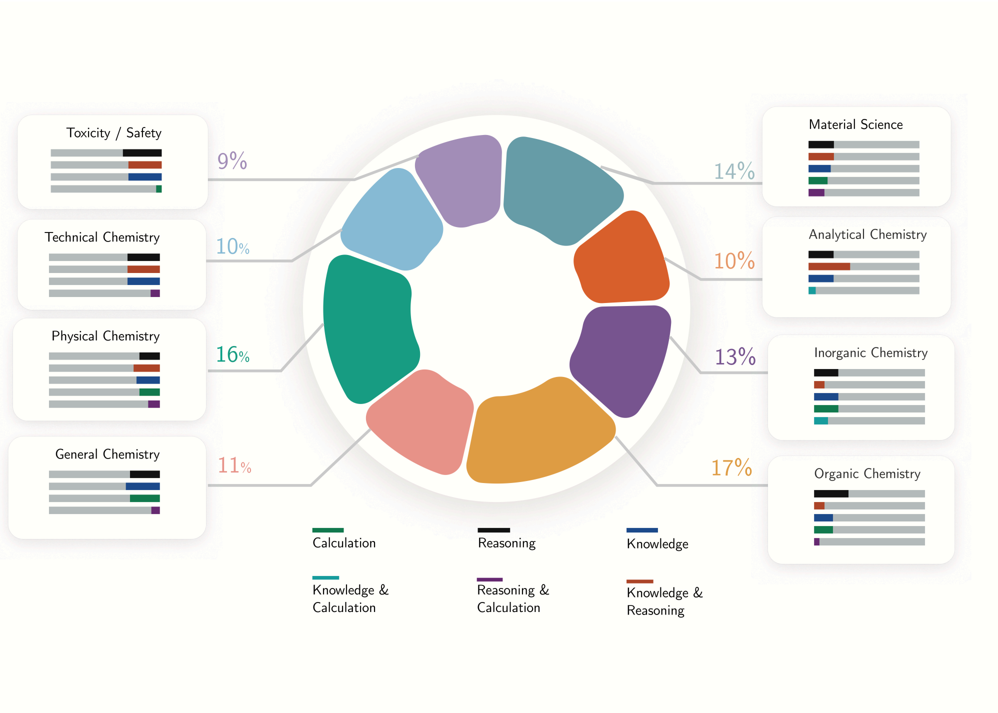

Supplementary Fig. 1 | Composition of the human subset. The circular plot shows the distribution of topics and required skills in the human subset of the ChemBench corpus. The human subset is a representative subset of the full corpus, with a balanced distribution of topics and skills.

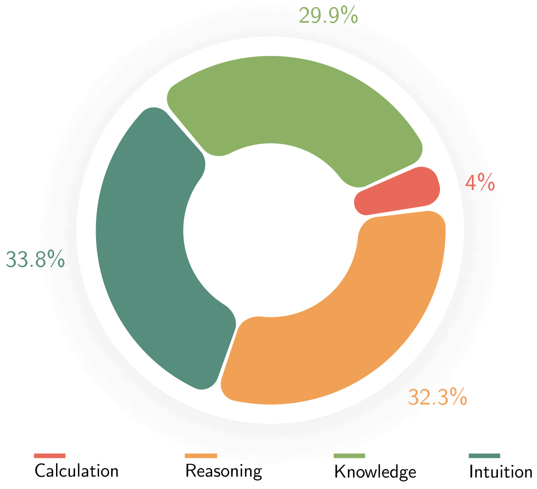

Supplementary Fig. 2 | Composition of the required skills considered in the ChemBench corpus. The circular plot shows the distribution of required skills in the ChemBench corpus.

In addition, as shown in Supplementary Fig. 2 the ChemBench corpus can be divided considering the different skills needed to solve the questions. The plot shows a balanced distribution of the required skills in the ChemBench corpus. Supplementary Table 2 shows an example question for each skill category.

Supplementary Table 2 | Examples for each required skill considered in the ChemBench corpus. The table shows the number of questions for each skill and an example question. Note that the total count in this table is bigger than the ChemBench corpus. This is because the same question can be annotated with two different skills, e.g., Reasoning and Calculation.

**Knowledge** 886 Questions
Which of the following salts is an acidic salt?<br>A. NH_4Cl<br>B. Na_2CO_3

C. NaH_2PO_4
D. Zn(OH)Cl

Reasoning 955 Questions

You make the following experimental observations: The sample consisted of small colorless/white crystals, wine-red transparent crystals, turquoise transparent crystals, and violet flakes. Most components dissolved in cold water, with turquoise crystals dissolving upon heating. Dimethylglyoxime produced a raspberry-red color with dissolved turquoise crystals. Violet flakes turned blue on contact and showed red/violet colors in flame tests. An oxidation melt turned green. No red coloration occurred with KSCN, but a blue organic phase formed with acetone. Potassium ferrocyanide showed no changes. KOH produced a brown/black precipitate. Alizarin-S testing resulted in red coloration after acidification. Heating with cobalt nitrate produced a bluish color without luster. Lead dioxide and sulfuric acid oxidation formed a violet solution. No changes occurred in chromate oxidation or titanium detection attempts. $BaCl_2$ formed precipitates in both original solution and soda extract. Lunge's reagent showed red coloration around zinc granules. Silver nitrate formed white precipitates that dissolved in NH_3 and reappeared with HNO_3 acidification. The separation procedure began with the aqueous solution being treated with $(NH_4)_2CO_3$ solution until a persistent turbidity appeared, followed by addition of dilute HCl until the turbidity disappeared. The pH was then adjusted to 5-6 with NH_3 (2 M) and heated on a water bath. Three spatula tips of urotropin were added, resulting in a white, rather flocculent precipitate. This precipitate was centrifuged off and washed with warm water. In the Urotropin group, the residue was dissolved in dilute HCl and diluted with water. An alkaline precipitation was performed, resulting in a brown coloration even after repeated washing of the urotropin precipitation. This precipitate dissolved in 2 M HCl, but showed no change when treated with either KSCN or potassium ferrocyanide. In the $(NH_4)_2S$ group, addition of dimethylglyoxim produced a very flocculent red precipitate that rose to the top of the test tube. When H_2S was introduced to the centrifugate, a black precipitation occurred. The precipitates did not completely dissolve in HCl. Treatment with KSCN resulted in a blue organic phase when layered with acetone. An alkaline precipitation produced a brown/black residue. No precipitation was observed when H_2S was introduced to the supernatant liquid after the alkaline precipitation. What ions are in the sample?
A. Al^{3+}
B. Co^{2+}
C. Cr^{3+}
D. Fe^{3+}
E. Mn^{2+}

F. Ni^{2+}
G. Zn^{2+}

**Intuition** 1001 Questions
Imagine an early virtual screening campaign setting (accounting for simple aspects such as oral availability and small molecular profile, but no other modalities such as covalency or bifunctionality). Which of the following two candidates would you prefer for further development?<br>A. [START_SMILES]CC1(C)Oc2ccc([N+](=O)[O-])cc2[C@@H](N2CCOCC2)[CH]1O[END_SMILES]<br>B. [START_SMILES]Cc1ccccc1N=C(S)N1CCC(NC(=O)c2ccco2)CC1[END_SMILES]

**Calculation** 118 Questions
Given that the average molar mass of the polymer chains in this sample of poly(lactic acid) (PLA) is $595\,\mathrm{g\,mol^{-1}}$ using end-group analysis, where 0.1619 g of PLA was dissolved in $25\,\mathrm{cm^3}$ of benzyl alcohol and titrated with $0.0400\,\mathrm{mol\,dm^{-3}}$ KOH solution. The volume of KOH solution required to reach the endpoint was $6.81\,\mathrm{cm^3}$. What is the average number of monomer units in each polymer chain of this sample?

Supplementary Note 3.3 Sources of semi-programatically generated questions

Supplementary Table 3 | Sources of semi-programatically generated questions. The table shows the sources and a brief description as well as the number of the semi-programatically generated questions.

source	description	question count
Number of isomers	MAYGEN[17] was used to compute the number of isomers for a set of SMILES extracted from the ZINC dataset[18]	24
Total electron count of molecules	Electron counts based on the data from `https://www.cheminfo.org/`	25
Oxidation states	Oxidation states questions based on the data from `https://www.cheminfo.org/`	10

Chemical reactivity	Questions are framed based on the information from the Cameo Chemicals website	276
Number of NMR signals	Molecules are sampled from the ZINC database[18], OpenChemLib[19] is used to compute the number of diasterotopically distinct hydrogen atoms	50
Point group of molecules	Our ChemCaption tool is used to assign the point group using spglib,[20] and then each case was manually checked to select well-defined cases	16
IUPAC-SMILES pairs	Sampled from the PubChem[21] database	10 + 10
PubChem[21] safety data	Daily allowable intakes according to the World Health Organization	10
	Definitions of hazard statements	10
	GHS classification of chemicals mined through the API	7
Safety	Materials' compatibility	20
	Chemical compatibility	296

Supplementary Note 4 Model performance

We also evaluated the model performance on the entire ChemBench corpus. Supplementary Fig. 3 shows the fraction of questions the models answered correctly.

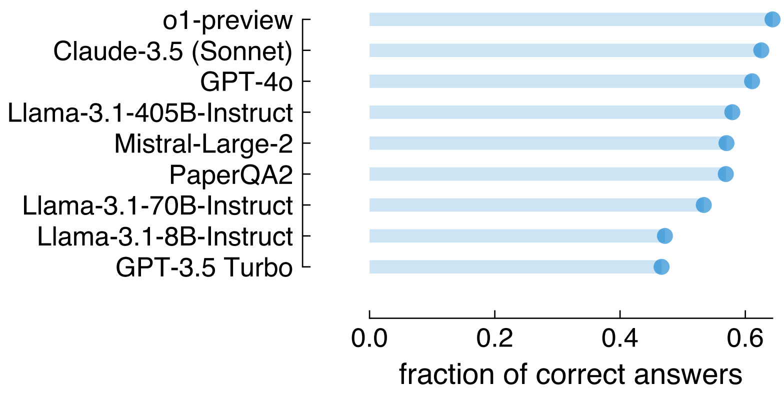

Supplementary Fig. 3 | Overall performance of the models on the ChemBench corpus. The bar plot shows the fraction of questions the models answered correctly. Scores computed on the entire ChemBench corpus.

Supplementary Fig. 4 shows the performance of the models on the different topics of the ChemBench corpus. The general pattern of performance varies significantly between the different topics and is also observed when the models are evaluated on the entire corpus. However, since some subjects are composed of questions from different sources, the ranking of the models is, in some instances, different from the one on ChemBench-Mini.

To further investigate the performance of the models, we also compared the performance on different data sources. Compared to topics, this is a more fine-grained analysis, as topics can be composed of questions from different sources. In Supplementary Fig. 5, we see that the performance of the models varies significantly between the different data sources. Interestingly, the performance of the models on questions sourced based on textbooks seems to be better for our models than some semi-programmatically created tasks, such as questions about the number of signals in an NMR spectrum.

Supplementary Fig. 6 shows the same analysis on ChemBench-Mini.

Supplementary Fig. 7 shows the performance of the models on the ChemBench corpus and the ChemBench-Mini subset. The relative performance difference between both is quite similar across most models. This makes ChemBench-Mini subset a reliable subset for human baseline comparison and particularly valuable for rapid prototyping and initial model assessment phases.

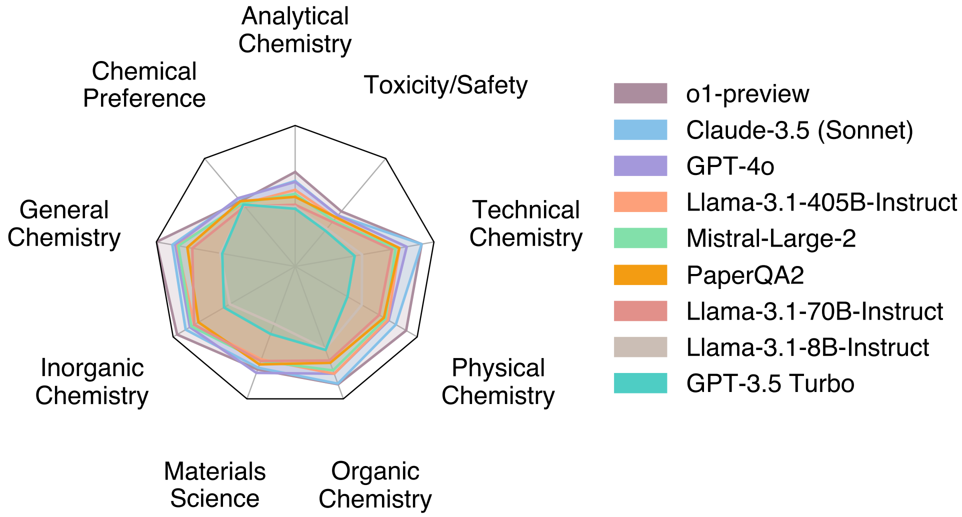

Supplementary Fig. 4 | Performance of the models on the different topics of the ChemBench corpus. The radar plot shows the performance of the models on the different topics of the ChemBench corpus. The performance is measured as the fraction of questions answered correctly by the models. A score of 1 (full coverage until the outer line of this plot) indicates that all questions were answered correctly, while a score of 0 indicates that none were answered correctly.

An interesting observation is the significant impact of chemical preference tasks on GPT-4's scores. A detailed breakdown of overall accuracy into scores on different skills and difficulty levels is provided in Supplementary Table 4 and Supplementary Table 5 for ChemBench corpus and the ChemBench-Mini subset, respectively.

Supplementary Table 4 | Performance of the models on the ChemBench corpus. The table shows the fraction of questions answered correctly by the models for different skills and difficulty levels. Models with "T-one" in the name were run for a temperature of 1, which allows us to study the temperature effect in the benchmark. Systems with "ReAct" in the name are tool augmented, i.e., they can call external tools such as web search or a calculator to answer the questions better. However, we limit those systems to ten calls to the LLM. This constraint often led the systems to not find the correct answer within the specified number of calls. In this case, we consider the answer as incorrect (see Supplementary Note 11).

Model	**Requires**				**Difficulty**			**Overall Accuracy**
	Calculation	Knowledge	Reasoning	Intuition	Basic	Intermediate	Advanced	
Claude-2	0.31	0.37	0.54	0.51	0.63	0.41	0.36	0.47
Claude-3 (Opus)	0.59	0.47	0.67	0.57	0.77	0.50	0.43	0.57
Claude-3.5 (Sonnet)	0.65	0.53	0.77	0.58	0.83	**0.55**	0.64	0.63
Claude-3.5 (Sonnet) React	0.71	0.50	0.77	**0.60**	0.81	0.54	0.75	0.62
Command-R+	0.19	0.35	0.50	0.51	0.55	0.40	0.20	0.45
Galactica-120b	0.02	0.02	0.03	0.00	0.04	0.00	0.00	0.02
Gemini-Pro	0.25	0.36	0.51	0.50	0.58	0.40	0.35	0.45
Gemma-1.1-7B-it	0.20	0.24	0.36	0.00	0.43	0.09	0.10	0.19
Gemma-1.1-7B-it-T-one	0.19	0.23	0.36	0.01	0.44	0.10	0.14	0.19
Gemma-2-9B-it	0.40	0.36	0.52	0.55	0.61	0.43	0.38	0.48
Gemma-2-9B-it-T-one	0.37	0.35	0.51	0.56	0.61	0.43	0.43	0.48
GPT-3.5 Turbo	0.25	0.35	0.53	0.53	0.60	0.42	0.36	0.47
GPT-4	0.48	0.46	0.65	0.16	0.74	0.28	0.57	0.41
GPT-4o	0.72	0.51	0.73	0.59	0.81	0.53	0.62	0.61
GPT-4o React	0.54	0.44	0.67	0.42	0.73	0.41	0.60	0.51
Llama-2-70B Chat	0.10	0.14	0.15	0.49	0.20	0.30	0.00	0.27
Llama-3-8B-Instruct	0.25	0.37	0.50	0.52	0.55	0.41	0.52	0.46
Llama-3-8B-Instruct-T-one	0.23	0.36	0.51	0.52	0.55	0.42	0.57	0.46
Llama-3-70B-Instruct	0.47	0.42	0.60	0.53	0.69	0.45	0.29	0.52
Llama-3-70B-Instruct-T-one	0.46	0.42	0.59	0.53	0.70	0.45	0.29	0.52
Llama-3.1-8B-Instruct	0.34	0.36	0.52	0.53	0.61	0.41	0.38	0.47
Llama-3.1-8B-Instruct-T-one	0.35	0.35	0.50	0.52	0.59	0.41	0.64	0.46
Llama-3.1-70B-Instruct	0.50	0.44	0.65	0.52	0.74	0.45	0.33	0.53
Llama-3.1-70B-Instruct-T-one	0.50	0.43	0.57	0.54	0.65	0.46	0.36	0.51
Llama-3.1-405B-Instruct	0.64	0.49	0.71	0.54	0.79	0.50	0.57	0.58
Mistral-Large-2	0.65	0.47	0.70	0.55	0.78	0.48	0.57	0.57
Mixtral-8x7b-Instruct	0.21	0.31	0.42	0.54	0.51	0.39	0.25	0.42
Mixtral-8x7b-Instruct-T-one	0.21	0.31	0.41	0.52	0.51	0.38	0.25	0.42
o1-preview	**0.80**	**0.57**	**0.80**	0.56	**0.86**	**0.55**	**0.85**	**0.64**
PaperQA2	0.62	0.49	0.66	0.56	0.75	0.49	0.57	0.57
Phi-3-Medium-4k-Instruct	0.34	0.36	0.53	0.53	0.61	0.42	0.50	0.47

Supplementary Table 5 | Performance of the models on ChemBench-Mini. The table shows the fraction of questions answered correctly by the models for different skills and difficulty levels.

Model	Requires			Difficulty			**Overall Accuracy**
	Calculation	Knowledge	Reasoning	Basic	Intermediate	Advanced	
Claude-2	0.29	0.52	0.51	0.57	0.45	0.36	0.49
Claude-3 (Opus)	0.62	0.64	0.67	0.78	0.61	0.43	0.67
Claude-3.5 (Sonnet)	0.62	0.75	0.75	0.79	0.70	0.64	0.73
Claude-3.5 (Sonnet) React	0.73	0.75	0.75	**0.83**	0.71	0.75	0.76
Command-R+	0.25	0.49	0.41	0.46	0.42	0.20	0.42
Galactica-120b	0.01	0.02	0.01	0.04	0.01	0.00	0.02
Gemini-Pro	0.27	0.49	0.46	0.50	0.43	0.35	0.45
Gemma-1.1-7B-it	0.23	0.40	0.36	0.42	0.33	0.10	0.35
Gemma-1.1-7B-it-T-one	0.21	0.39	0.35	0.41	0.31	0.14	0.34
Gemma-2-9B-it	0.44	0.46	0.45	0.56	0.44	0.40	0.49
Gemma-2-9B-it-T-one	0.40	0.44	0.42	0.55	0.41	0.43	0.46
GPT-3.5 Turbo	0.31	0.46	0.45	0.51	0.41	0.36	0.44
GPT-4	0.47	0.63	0.64	0.67	0.62	0.57	0.64
GPT-4o	0.68	0.72	0.74	0.81	0.67	0.65	0.72
GPT-4o React	0.60	0.59	0.63	0.69	0.57	0.60	0.62
Human	0.28	0.23	0.27	0.32	0.24	0.27	0.27
Llama-2-70B Chat	0.12	0.18	0.13	0.19	0.13	0.00	0.14
Llama-3-8B-Instruct	0.26	0.54	0.44	0.43	0.45	0.50	0.44
Llama-3-8B-Instruct-T-one	0.28	0.54	0.45	0.43	0.46	0.57	0.45
Llama-3-70B-Instruct	0.49	0.63	0.57	0.67	0.59	0.30	0.60
Llama-3-70B-Instruct-T-one	0.49	0.61	0.57	0.67	0.57	0.29	0.59
Llama-3.1-8B-Instruct	0.33	0.47	0.44	0.55	0.39	0.40	0.46
Llama-3.1-8B-Instruct-T-one	0.38	0.47	0.40	0.51	0.38	0.64	0.44
Llama-3.1-70B-Instruct	0.52	0.62	0.63	0.73	0.62	0.35	0.64
Llama-3.1-70B-Instruct-T-one	0.50	0.55	0.58	0.66	0.54	0.36	0.57
Llama-3.1-405B-Instruct	0.66	0.69	0.70	0.72	0.69	0.57	0.69
Mistral-Large-2	0.66	0.65	0.68	0.72	0.66	0.60	0.68
Mixtral-8x7b-Instruct	0.23	0.40	0.37	0.45	0.32	0.25	0.36
Mixtral-8x7b-Instruct-T-one	0.21	0.39	0.35	0.46	0.29	0.25	0.36
o1-preview	**0.77**	**0.79**	**0.81**	0.82	**0.77**	**0.85**	**0.80**
PaperQA2	0.63	0.69	0.65	0.70	0.67	0.55	0.67
Phi-3-Medium-4k-Instruct	0.34	0.49	0.52	0.57	0.47	0.50	0.51

Supplementary Table 6 | Performance of the models on the ChemBench corpus. The table shows the fraction of questions answered correctly by the models for different topics. Models with "T-one" in the name were run for a temperature of 1, which allows us to study the temperature effect in the benchmark. Systems with "ReAct" in the name are tool augmented, i.e., they can call external tools such as web search or a calculator to answer the questions better. However, we limit those systems to ten calls to the LLM. This constraint often led the systems to not find the correct answer within the specified number of calls. In this case, we consider the answer as incorrect (see Supplementary Note 11).

Model	Analytical	Chemical Preference	General	Inorganic	Materials Science	Organic	Physical	Technical	Toxicity/Safety	Overall Accuracy
Claude-2	0.39	0.51	0.51	0.56	0.30	0.60	0.54	0.47	0.33	0.47
Claude-3 (Opus)	0.48	0.57	0.77	0.75	0.50	0.69	0.69	0.70	0.41	0.57
Claude-3.5 (Sonnet)	0.57	0.58	0.83	0.82	0.50	0.82	0.81	**0.85**	0.44	0.63
Claude-3.5 (Sonnet) React	0.58	**0.60**	0.87	0.79	0.50	**0.83**	0.80	0.80	0.41	0.62
Command-R+	0.36	0.51	0.49	0.50	0.30	0.56	0.37	0.50	0.31	0.45
Galactica-120b	0.00	0.00	0.05	0.05	0.00	0.00	0.06	0.00	0.02	0.02
Gemini-Pro	0.41	0.50	0.49	0.44	0.50	0.58	0.46	0.47	0.31	0.45
Gemma-1.1-7B-it	0.21	0.00	0.33	0.43	0.40	0.39	0.34	0.38	0.23	0.19
Gemma-1.1-7B-it-T-one	0.21	0.01	0.35	0.41	0.40	0.38	0.35	0.38	0.22	0.19
Gemma-2-9B-it	0.32	0.55	0.55	0.53	0.50	0.57	0.54	0.53	0.34	0.48
Gemma-2-9B-it-T-one	0.30	0.56	0.56	0.51	0.40	0.57	0.53	0.47	0.34	0.48
GPT-3.5 Turbo	0.40	0.53	0.49	0.53	0.50	0.60	0.44	0.40	0.31	0.47
GPT-4	0.44	0.16	0.70	0.68	0.50	0.68	0.69	0.70	0.41	0.41
GPT-4o	0.56	0.59	0.81	0.80	0.60	0.75	0.76	0.75	0.44	0.61
GPT-4o React	0.47	0.42	0.76	0.73	0.40	0.72	0.62	0.72	0.37	0.51
Llama-2-70B Chat	0.07	0.49	0.13	0.23	0.20	0.15	0.18	0.12	0.14	0.27
Llama-3-8B-Instruct	0.43	0.52	0.45	0.48	0.40	0.57	0.40	0.60	0.32	0.46
Llama-3-8B-Instruct-T-one	0.42	0.52	0.44	0.52	0.30	0.55	0.38	0.62	0.32	0.46
Llama-3-70B-Instruct	0.43	0.53	0.61	0.64	0.60	0.65	0.64	0.62	0.37	0.52
Llama-3-70B-Instruct-T-one	0.39	0.53	0.61	0.66	0.60	0.64	0.65	0.60	0.37	0.52
Llama-3.1-8B-Instruct	0.41	0.53	0.51	0.50	0.40	0.58	0.55	0.45	0.33	0.47
Llama-3.1-8B-Instruct-T-one	0.38	0.52	0.53	0.48	0.40	0.58	0.48	0.40	0.32	0.46
Llama-3.1-70B-Instruct	0.42	0.52	0.69	0.74	0.50	0.68	0.68	0.65	0.38	0.53
Llama-3.1-70B-Instruct-T-one	0.36	0.54	0.67	0.67	0.50	0.58	0.55	0.55	0.39	0.51
Llama-3.1-405B-Instruct	0.52	0.54	0.79	0.78	0.70	0.74	0.73	0.70	0.42	0.58
Mistral-Large-2	0.50	0.55	0.79	0.77	0.40	0.73	0.73	0.68	0.40	0.57
Mixtral-8x7b-Instruct	0.28	0.54	0.43	0.51	0.40	0.50	0.37	0.33	0.27	0.42
Mixtral-8x7b-Instruct-T-one	0.28	0.52	0.45	0.50	0.40	0.50	0.39	0.33	0.27	0.42
o1-preview	**0.62**	0.56	**0.93**	**0.90**	**0.80**	0.82	**0.89**	**0.85**	**0.48**	**0.64**
PaperQA2	0.48	0.56	0.73	0.71	0.60	0.67	0.73	0.70	0.42	0.57
Phi-3-Medium-4k-Instruct	0.35	0.53	0.48	0.58	0.40	0.56	0.49	0.55	0.33	0.47

Supplementary Table 7 | Performance of the models on ChemBench-Mini. The table shows the fraction of questions answered correctly by the models for different topics.

Model	Analytical	General	Inorganic	Materials Science	Organic	Physical	Technical	Toxicity/Safety	Overall Accuracy
Claude-2	0.77	0.46	0.47	0.00	0.62	0.42	0.52	0.60	0.49
Claude-3 (Opus)	0.77	0.73	0.76	0.50	0.62	0.62	0.74	0.65	0.67
Claude-3.5 (Sonnet)	0.86	0.69	0.68	0.50	0.79	**0.79**	0.87	0.55	0.73
Claude-3.5 (Sonnet) React	0.77	0.81	0.71	0.50	**0.88**	**0.79**	0.83	0.55	0.76
Command-R+	0.45	0.35	0.44	0.00	0.33	0.25	0.57	0.60	0.42
Galactica-120b	0.00	0.00	0.03	0.00	0.00	0.12	0.00	0.05	0.02
Gemini-Pro	0.55	0.38	0.41	0.00	0.58	0.46	0.43	0.45	0.45
Gemma-1.1-7B-it	0.36	0.31	0.44	0.50	0.46	0.29	0.39	0.40	0.35
Gemma-1.1-7B-it-T-one	0.32	0.31	0.41	0.50	0.42	0.33	0.39	0.40	0.34
Gemma-2-9B-it	0.45	0.54	0.47	0.50	0.54	0.58	0.61	0.50	0.49
Gemma-2-9B-it-T-one	0.45	0.50	0.44	0.50	0.58	0.54	0.52	0.45	0.46
GPT-3.5 Turbo	0.55	0.35	0.44	0.50	0.58	0.50	0.43	0.55	0.44
GPT-4	0.77	0.69	0.59	**1.00**	0.79	0.67	0.83	0.60	0.64
GPT-4o	0.73	0.69	0.71	0.50	0.75	0.75	0.78	0.60	0.72
GPT-4o React	0.55	0.58	0.68	0.50	0.75	0.58	0.74	0.45	0.62
Human	0.31	0.41	0.30	0.24	0.36	0.26	0.20	0.22	0.27
Llama-2-70B Chat	0.00	0.19	0.15	0.50	0.21	0.17	0.13	0.25	0.14
Llama-3-8B-Instruct	0.68	0.27	0.44	0.00	0.50	0.29	0.61	0.50	0.44
Llama-3-8B-Instruct-T-one	0.64	0.27	0.53	0.00	0.46	0.25	0.65	0.50	0.45
Llama-3-70B-Instruct	0.64	0.62	0.62	0.50	0.75	0.58	0.74	0.50	0.60
Llama-3-70B-Instruct-T-one	0.59	0.62	0.62	0.50	0.71	0.58	0.70	0.60	0.59
Llama-3.1-8B-Instruct	0.59	0.35	0.53	0.00	0.50	0.50	0.52	0.60	0.46
Llama-3.1-8B-Instruct-T-one	0.59	0.38	0.47	0.50	0.50	0.42	0.39	0.55	0.44
Llama-3.1-70B-Instruct	0.59	0.62	0.74	0.50	0.75	0.71	0.70	0.60	0.64
Llama-3.1-70B-Instruct-T-one	0.45	0.58	0.59	0.50	0.67	0.62	0.65	0.45	0.57
Llama-3.1-405B-Instruct	0.68	0.73	0.74	**1.00**	0.71	0.75	0.70	0.60	0.69
Mistral-Large-2	0.82	0.65	0.68	**1.00**	0.75	0.71	0.70	0.50	0.68
Mixtral-8x7b-Instruct	0.41	0.35	0.47	0.50	0.33	0.33	0.30	0.40	0.36
Mixtral-8x7b-Instruct-T-one	0.45	0.42	0.41	0.50	0.38	0.38	0.30	0.35	0.36
o1-preview	**0.91**	**0.92**	**0.79**	**1.00**	0.79	**0.79**	**0.91**	**0.70**	**0.80**
PaperQA2	0.68	0.73	0.62	**1.00**	0.58	0.71	0.78	0.65	0.67
Phi-3-Medium-4k-Instruct	0.45	0.42	0.56	0.50	0.62	0.54	0.57	0.50	0.51

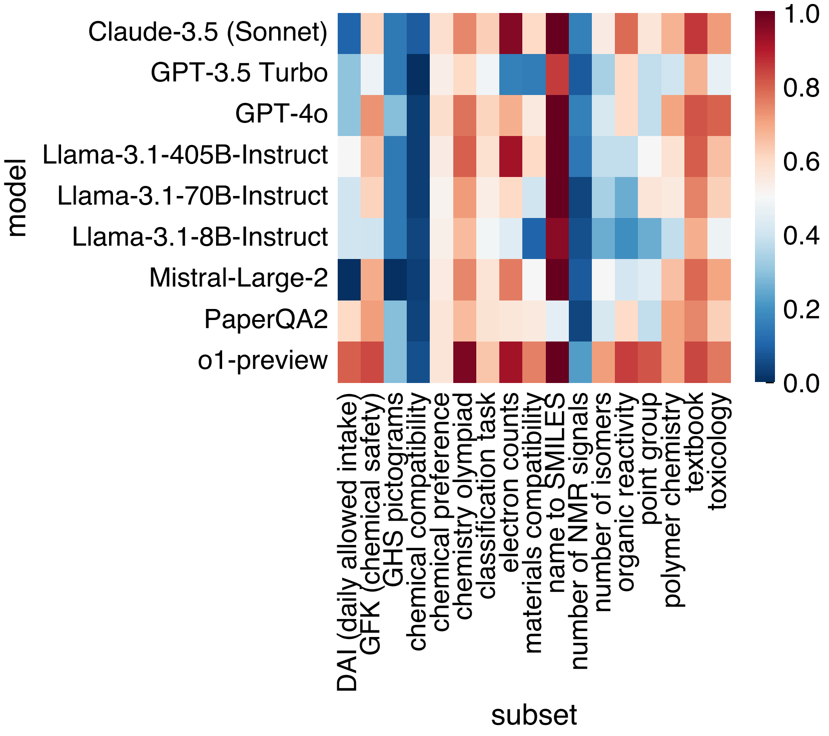

Supplementary Fig. 5 | Fraction of correctly answered questions per data source on ChemBench-Mini. The heatmap shows, in color, the fraction of questions answered correctly by different systems for some of our data sources. The performance is measured as the fraction of questions answered correctly by the models. A score of one (red) indicates that all questions were answered correctly, while a score of zero (blue) indicates that none of the questions were answered correctly. We see that the performance of the models varies significantly between the different data sources. For instance, it is interesting to observe that questions sourced based on textbooks seem easier for our leading models than for humans. However, this performance does not correlate with performance on other sources, e.g., semi-programmatically created tasks such as questions about the number of signals in an NMR spectrum.

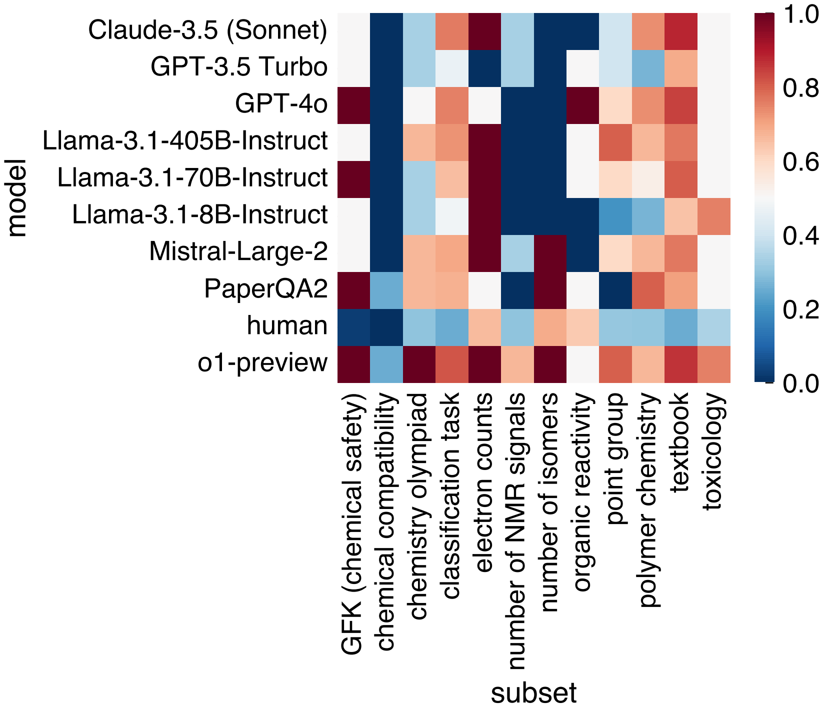

Supplementary Fig. 6 | Fraction of correctly answered questions per data source on ChemBench-Mini. The heatmap shows, in color, the fraction of questions answered correctly by different systems for some of our data sources. The performance is measured as the fraction of questions answered correctly by the models. A score of one (red) indicates that all questions were answered correctly, while a score of zero (blue) indicates that none were answered correctly. We see that the performance of the models varies significantly between the different data sources. For instance, it is interesting to observe that questions sourced based on textbooks seem easier for the leading models than for humans. However, this performance does not correlate with performance on other sources, e.g., semi-programmatically created tasks such as questions about the number of signals in an NMR spectrum.

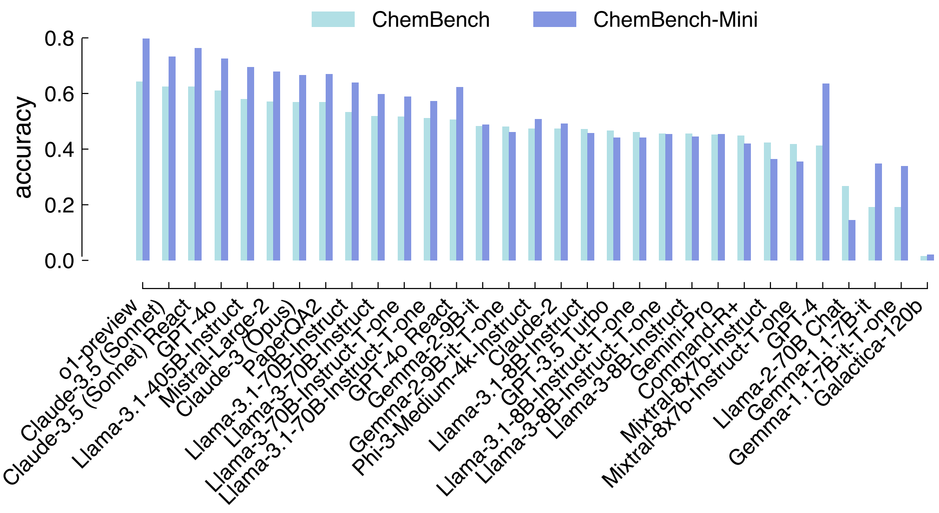

Supplementary Fig. 7| Performance of the models on the ChemBench corpus and ChemBench-Mini subset. The bar plot shows the fraction of questions that were answered completely correctly, highlighting the relative performance of different models on both the ChemBench corpus and the ChemBench-Mini subset. We see that the model ranking remains fairly consistent across both sets

Supplementary Note 5 Performance as a function of molecular features

To better understand whether the performance of the models is correlated with specific features of the molecules, we analyzed the performance of the models as a function of the number of atoms and the complexity of the molecules. Supplementary Fig. 8 shows that the performance of the models is not correlated with the complexity of the molecules but rather with the number of atoms (Supplementary Fig. 9, similar trivial correlation for Supplementary Fig. 10).

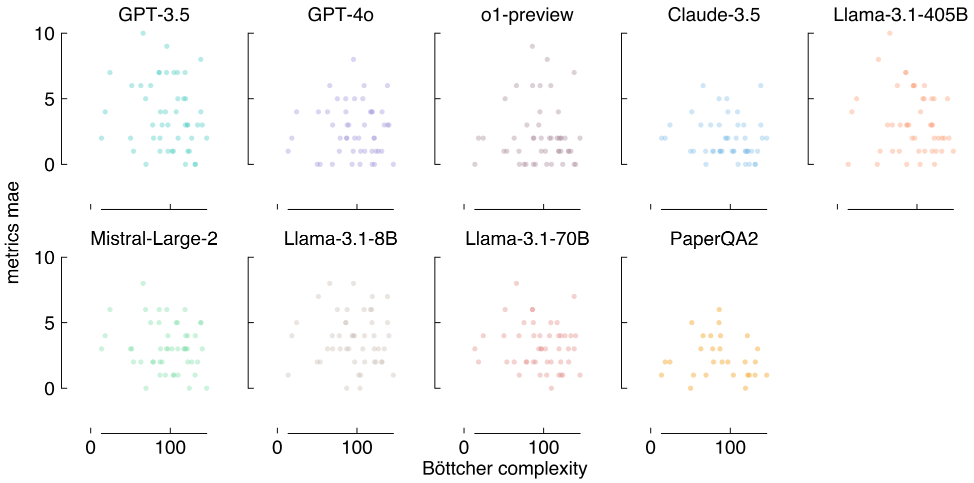

Supplementary Fig. 8 | Dependence of the mean absolute error in predicting the number of NMR signals on the Böttcher complexity of the molecules. The complexity measure proposed by Böttcher[22] is an information-theoretic additive measure of compound complexity that follows chemical intuitions. The plot shows that for the LLMs, the predictive performance (measured as the mean absolute error in the prediction of the number of NMR signals) is not correlated with the complexity of the molecules (that is, molecules tend to not to be able to predict the number of NMR signals regardless of molecular complexity). For inference based on reasoning, one would expect that the complexity of the molecule is a good predictor of the difficulty of the question.

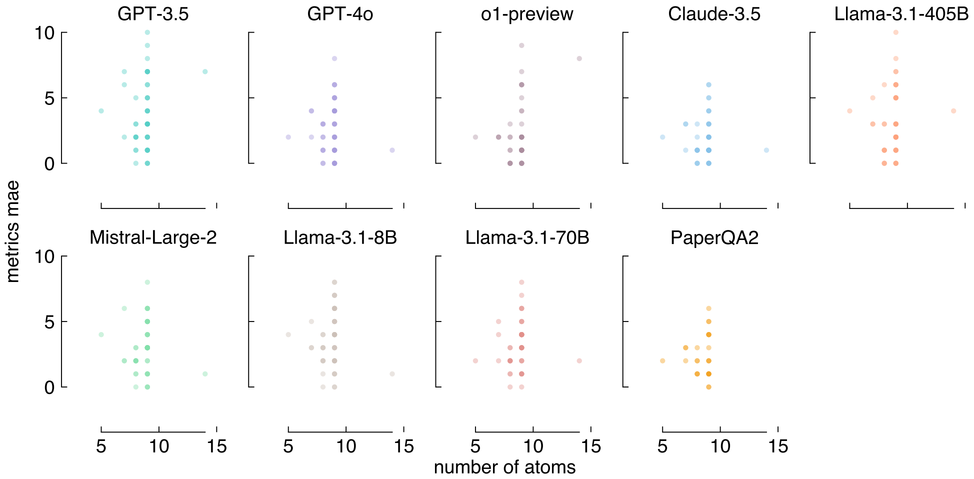

Supplementary Fig. 9 | Dependence of the mean absolute error in predicting the number of NMR signals on the number of atoms.

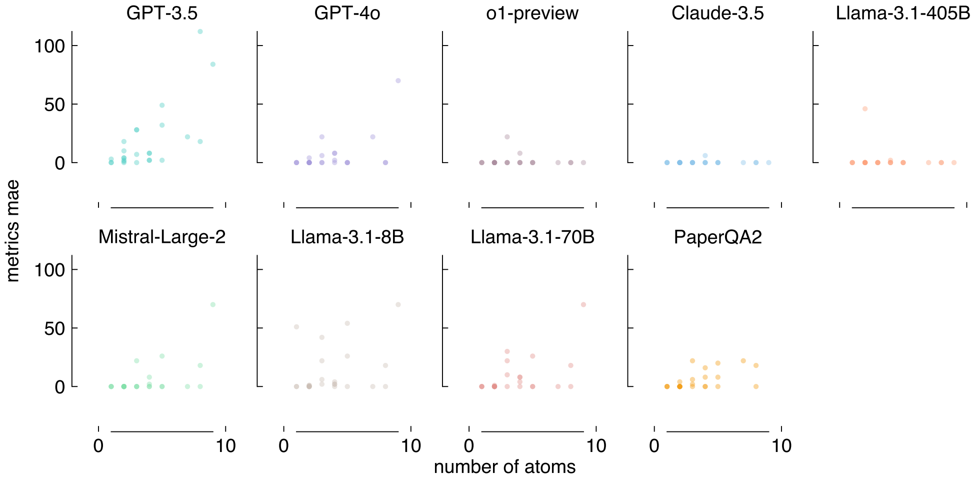

Supplementary Fig. 10 | Dependence of the mean absolute error in predicting total electron counts on the number of atoms. The plot shows that for the LLMs, the predictive performance (measured as the mean absolute error in the prediction of the total electron counts) is sometimes correlated with the number of atoms in the molecule.

Supplementary Note 6 Influence of model scale

Supplementary Fig. 11 shows the performance of the models as a function of the number of parameters in the model. We see that the performance of the models correlates with their size for the models of the LLama-3 and Llama-3.1 herd of models.

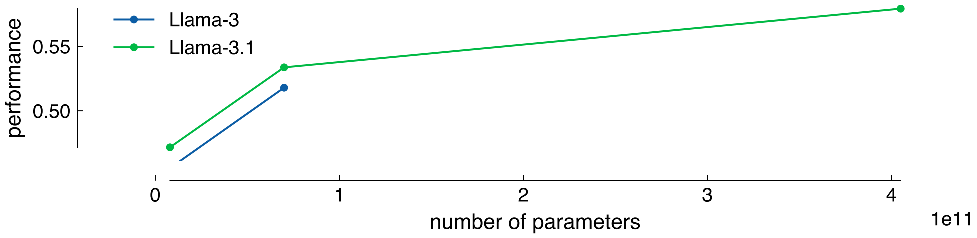

Supplementary Fig. 11 | Performance of models as a function of model size. The plot shows the performance of the models as a function of the parameter count. The performance is measured as the fraction of questions answered correctly by the models. We see that the performance of the models correlates with scale for the models of the LLama-3 and Llama-3.1 herd of models.

Supplementary Note 7 Refusal detection

LLMs typically undergo refusal training to prevent harmful or undesirable outputs. As a result, models may decline to answer questions perceived as potentially adversarial prompts. To automatically detect refusals, we use a modified regular expression reported by LLM Guard[23] to detect commonly used refusal phrases.

Supplementary Table 8 lists how many refusals were detected for the responses of different models on ChemBench. Overall, we find that refusals do not majorly affect the performance measured by ChemBench.

Supplementary Note 8 LLM Parsing

Our parsing workflow uses pipelines based on regular expressions to extract the answers. In some cases, however, the answers are not directly extractable from the responses, for instance, when the model does not follow the formatting instructions. In these cases, we use a fallback mechanism to extract the answers. The fallback mechanism uses an LLM to extract the answers from the responses. The LLM is provided with the response and the question and is prompted to only extract but not generate the answer. We used Llama-3-70B-Instruct, accessed via the Groq API. Supplementary Table 8 tabulates the number of times the fallback mechanism was used for each model.

Supplementary Table 8 | Refusal counts and parsing. The table shows the number of refusals detected and the number of times the LLM fallback parsing mechanism was used for each model.

Model	**Refusal**		**LLM Extraction**	
	N° of Questions	Fraction	N° of Questions	Fraction
Claude 2	100	0.035868	0	0.000000
Claude 3	9	0.003228	0	0.000000
Claude 3.5 Sonnet	31	0.011119	3	0.001076
Claude-3.5 ReAct	0	0.000000	129	0.046270
Command R+	0	0.000000	5	0.001793
Gemini Pro	0	0.000000	4	0.001435
Gemma 1.1 7B	0	0.000000	18	0.006456
Gemma 1.1 7B Temp=1	1	0.000359	22	0.007891
Gemma 2 9B	5	0.001793	25	0.008967
Gemma 2 9B Temp=1	5	0.001793	37	0.013271
GPT-3.5 Turbo	0	0.000000	8	0.002869
GPT-4	0	0.000000	3	0.001076
GPT-4o	0	0.000000	7	0.002511
GPT-4o ReAct	0	0.000000	667	0.239240
Llama 2 70B Chat	1	0.000359	3	0.001076
Llama 3 8B	0	0.000000	9	0.003228
Llama 3 8B Temp=1	6	0.002152	11	0.003945
Llama 3 70B	0	0.000000	16	0.005739
Llama 3 70B Temp=1	1	0.000359	17	0.006098
Llama 3.1 8B	0	0.000000	35	0.012554
Llama 3.1 8B Temp=1	3	0.001076	30	0.010760
Llama 3.1 70B	0	0.000000	53	0.019010
Llama 3.1 70B Temp=1	1	0.000359	245	0.087877
Llama 3.1 405B	0	0.000000	22	0.007891
Mistral Large 2 123B	0	0.000000	2	0.000717
Mixtral 8x7B	4	0.001435	54	0.019369
Mixtral 8x7B Temp=1	7	0.002511	58	0.020803
o1-preview	0	0.000000	2	0.000717
Paper QA	35	0.012554	52	0.018651
Phi 3 Medium 4K	0	0.000000	7	0.002511

Supplementary Note 9 Implementation

An overview of the benchmarking pipeline implemented in ChemBench is shown in Supplementary Fig. 12. More detailed information can be found in the online documentation of the ChemBench package at `https://lamalab-org.github.io/chembench/`.

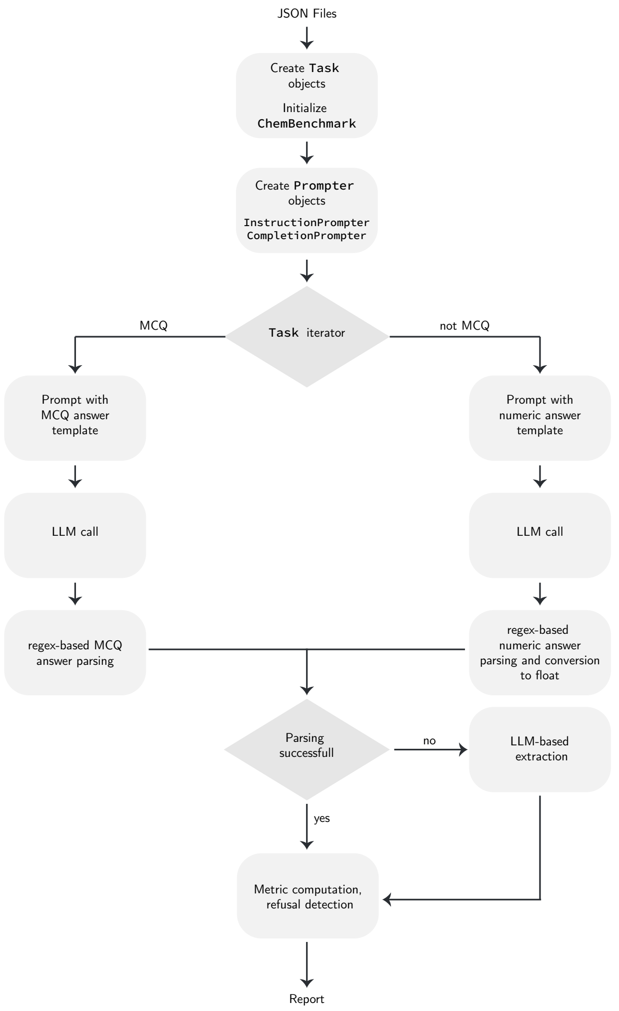

Supplementary Fig. 12 | Overview of the benchmarking pipeline implemented in ChemBench. The process begins with JSON files containing task data, which are used to create `Task` objects and initialize the `ChemBenchmark`. `Prompter` objects are then created to handle different types of prompts for instruction-tuned and completion models. The `Task` Iterator differentiates between MCQ and other question types. For each task type, appropriate prompts are generated and passed to the LLM. The responses are then processed using regex-based parsing methods specific to MCQ or numeric answers (after obtaining the relevant part of the response from the instruction-tuned models). The regex-based parsing is elaborate and can handle special cases such as scientific notation or Roman numerals. If the initial parsing is unsuccessful, the system employs an LLM-based extraction method as a fallback. The parsed or extracted answers then undergo metric computation and refusal detection.

Supplementary Note 10 Human baseline

App To facilitate the collection of responses, we developed a responsive web application in Typescript using the Next.js[24] app router framework. This application handles serving the user interface and exposes various representational state transfer (REST) APIs for relevant operations. We utilize a Postgresql. The web application is styled with Tailwind CSS[25] using the shadcn/ui component library and uses NextAuth[26] for easy and secure user authentication. The application is hosted on the Vercel web hosting platform.

In the applications, human participants were presented with molecules as rendered drawings and SMILES strings. LaTeX equations and chemical equations were rendered using MathJax (Supplementary Fig. 13).

Statistics Supplementary Fig. 14 shows the distribution of scores our human scorers achieved.

We also recorded the time humans took to answer the questions (Supplementary Fig. 15). This time is the time from the question being displayed to the human to the human submitting the answer.

Additionally, we prompted users to provide additional information about their experience in chemistry. While we recorded fine-grained information, e.g., their specialization, we focused on the number of years since the first university-level chemistry course. Supplementary Fig. 16 shows that the experience of the human scorers was weakly correlated with the correctness of their answers (Supplementary Fig. 16, Spearman's $\rho \approx 0.19$, and $p \approx 0.31$).

Tool use In our study, humans were allowed to use tools for answering some questions. They could also report what tools they used for answering questions. As Supplementary Fig. 17 shows, the most common tool was some form of web search (which, according to the free text responses, often was a multi-step process).

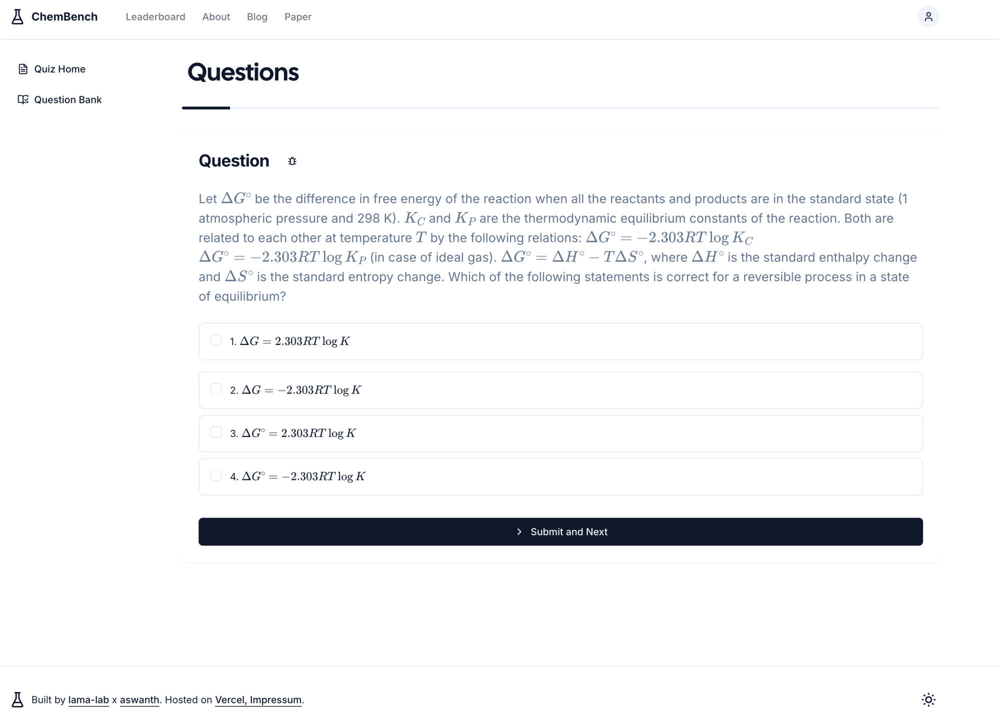

(a) A physcial chemistry question.

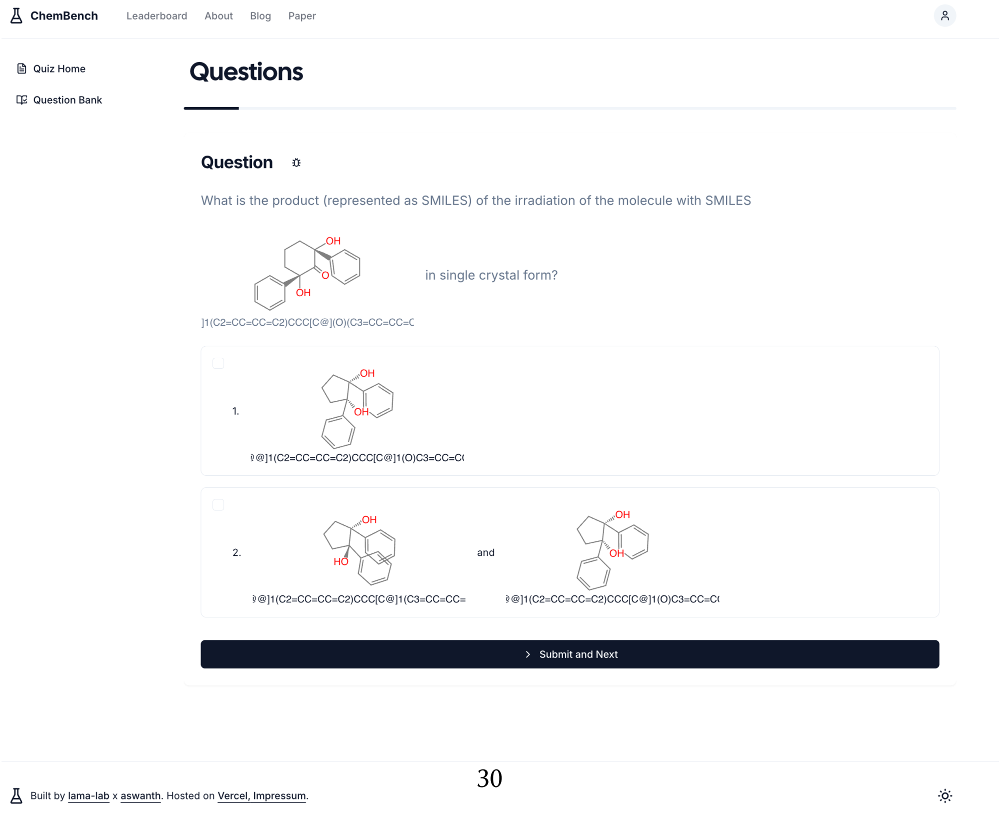

(b) An organic chemistry question.

Supplementary Fig. 13 | Examples of how questions were shown to the human participants.

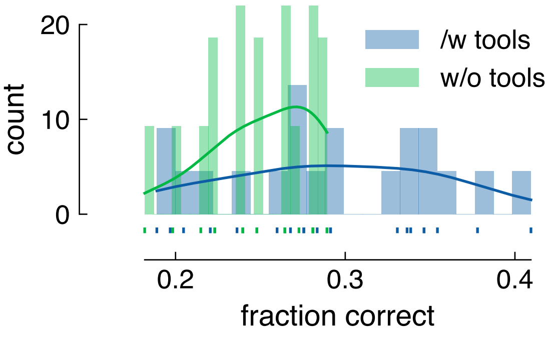

Supplementary Fig. 14 | Distribution of human scores. The histogram and kernel density estimates show the fraction of questions answered correctly by the human volunteers. Since the best possible score for each question is one and the worst possible score is zero, the values on this plot are between zero and one. A score of one would mean that a volunteer answered all questions correctly. A score of zero would mean that no question was answered correctly. We find that the scores for the questions that the human volunteers answered with tools are generally lower than the scores for the questions that the human volunteers answered without tools.

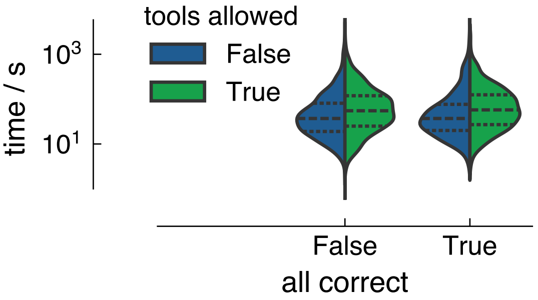

Supplementary Fig. 15 | Time taken by human scorers to answer questions vs. correctness of their answers. From the plot, it is clear that there is no clear dependence of the correctness of the answers on the time taken by the human scorers to answer the questions. However, we see that human scorers typically took longer to correctly answer questions with tool use.

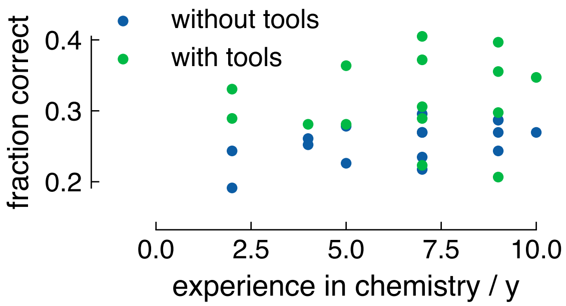

Supplementary Fig. 16 | Experience of human scorers vs. correctness of their answers. The experience (in the number of years since the first university-level chemistry course) of the human scorers wasp correlated with the correctness of their answers.

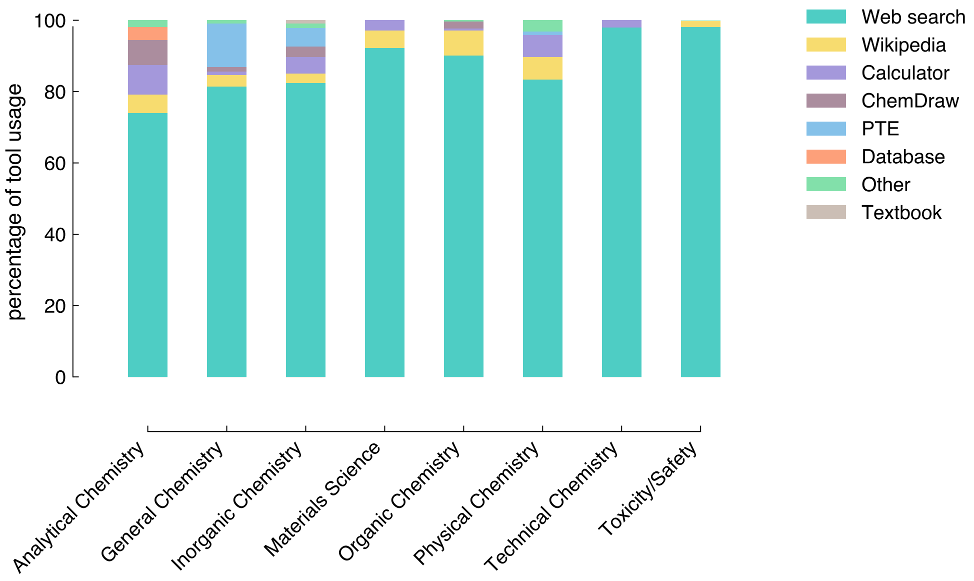

Supplementary Fig. 17 | Tool usage by human scorers. The plot shows the most commonly used tools by human participants.

Supplementary Note 11 Tool augmented models

In addition to directly prompting LLMs, we also investigated the performance of tool-augmented systems. For this, we investigated the two models that showed the best overall results, GPT-4o and Claude-3.5 (Sonnet) (o1-preview overperformed both models but is not recommended using this model with such "reasoning" prompts such as ReAct[10] or chain of thought (CoT)[27]). For both models, we created a ReAct-style tool augmentation environment in which models had access to WolframAlpha, the ArXiv API, Wikipedia, and web search (using Brave search API). We based the selection of these tools on the most used tools by humans (see Supplementary Fig. 17). Additionally, we added two specific tools to convert International Union of Pure and Applied Chemistry (IUPAC) names to SMILES, and SMILES to IUPAC names. These conversion tools allow us to understand better how the agents perform for specific questions in this agent environment configuration. We implemented the systems using Langchain[28] with the default ReAct prompt and constrained the system to a maximum of ten LLM calls.

While for Claude-3.5 (Sonnet) the overall performance did not change, we observe a decrease in performance for GPT-4o compared with the LLM without tools (Supplementary Table 4). If we study the results by each type of question, we observe an improvement for questions regarding electron counts or point groups of compounds. However, the scores decreased for questions about the number of isomers or globally harmonized system of classification and labelling of chemicals (GHS) pictograms. For the specific questions about converting IUPAC names to SMILES, the results decreased notably despite the models having access to specific tools prepared for those questions. By studying the reasoning path for these cases, we found that the error results from the models responding in a format that the LangChain framework with the default ReAct loop cannot handle. This indicates that agent frameworks need optimization to be more robust. It involves not only equipping the LLMs with tools but also necessitates engineering efforts to create robust systems.

Supplementary Note 12 Confidence estimates

Since it is important to understand if models can provide an indication of whether their answer might likely be incorrect, we prompted some of our top performing LLMs to return the confidence in providing a correct answer on an ordinal scale. This is similar to the verbalized confidence scores reported by Xiong et al.[29]. We find that the models show different distributions of confidence scores, which, for some, are skewed to the extremes.

In addition, we also analyzed the confidence estimated via the log probabilities of the answer tokens. This probability of a token given the context is not necessarily the same as the confidence in the correctness of the answer. However, it is still often used as a proxy.

Our analysis of both log probabilities and prompting confidence reveals distinct calibration behaviors across different language models. GPT-4o demonstrates an overconfident tendency, often assigning high probabilities even to incorrect answers. However, when GPT-4o displays high confidence, it accurately predicts correct answers approximately $80\,\%$ of the time. In contrast, Llama-3.1-8B-Instruct confidence distribution is more evenly spread, with a majority of predictions centered around 0.5. High-confidence predictions from Llama-3.1-8B-Instruct are less frequent compared to GPT-4o, and unlike GPT-4o, high confidence does not necessarily correlate with a higher chance of correct answers.

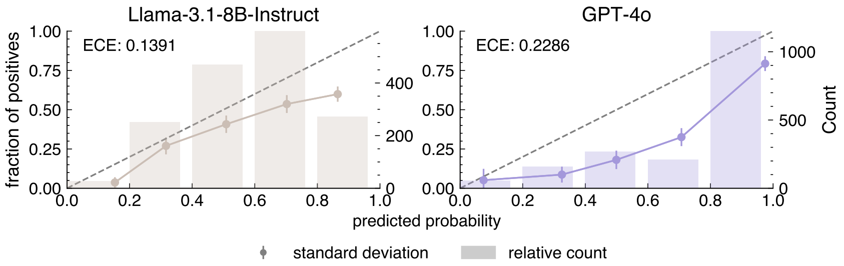

Supplementary Fig. 18 | Reliability diagram of logit-based confidence estimates. For this analysis, we obtained the linear probability from the logprobs of the models. Only logprobs of the tokens corresponding to the answers were considered. Linear probability was computed by taking the exponential of logprobs (for sequences with multiple tokens, values were multiplied). The plot shows the average predicted probability and the fraction of correct answers for each bin of linear probabilities. The ideal scenario is a diagonal line, indicating perfect calibration where the model's confidence aligns perfectly with the actual correctness. The error bars indicate the standard deviation (the number of samples per bin is given by the bar height). The expected calibration error (ECE) value quantifies the overall calibration performance, with a lower ECE indicating better calibration.

Supplementary Note 13 Impact of sampling temperature

We also investigated the impact of sampling temperature (i.e. temperature 0 means always sampling the most likely token, higher temperatures introduce some randomness in the generation process) on the performance of the models. Supplementary Fig. 19 shows that, generally, the performance of models tends to decrease with increasing temperature.

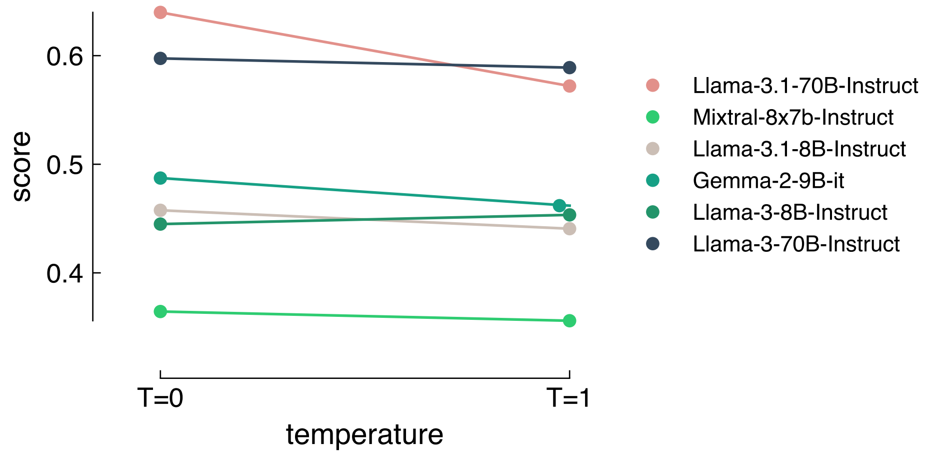

Supplementary Fig. 19 | Impact of sampling temperature on the performance of the models. The plot shows the performance of the models at zero temperature (i.e., greedy decoding) and temperature of one. The performance is measured in terms of the fraction of questions answered correctly.

Supplementary Note 14 Summary of trends and recommendations

o1-preview consistently leads across both the ChemBench corpus and ChemBench-Mini. Claude-3.5 (Sonnet), GPT-4o, Llama-3.1-405B-Instruct form the next tier of strong performers. These models show robust performance across all skills and difficulty levels.

Larger models generally perform better (Llama-3.1-405B-Instruct > Llama-3.1-70B-Instruct > Llama-3.1-8B-Instruct). However, smaller but well-designed models can compete with larger models. For example, Gemma-2-9B-it has an overall accuracy of 0.48 on the ChemBench corpus, which is only 10% less accurate than the much larger Llama-3.1-405B-Instruct model.

One can observe a clear progression of performance across model families (e.g., Claude-3.5 (Sonnet) > Claude-3 > Claude-2 or GPT-4o > GPT-4 > GPT-3.5 Turbo Zero-T). Newer versions consistently outperform their predecessors. Scores on knowledge-intensive tasks are typically lower than those on calculation and reasoning-intensive questions.

Based on these findings, some scope for improving these models could be to focus on enhancing knowledge-based training, possibly through improved pre-training on chemistry-specific texts or with chemistry knowledge-base integration. However, innovation is also needed to incorporate this information into the systems. For example, even PaperQA2 could not outperform the LLM it is used for directing the tools, even though the agent has access to literature evidence. This suggests we might need to not only focus on building chemistry-specific retrieval datasets since current systems fail to retrieve the relevant papers and should be coupled with more domain-specific databases. However, the observation that our ReAct agents were too fragile to answer questions for which they had custom-made tools available correctly suggests that we also must invest in building more robust agent frameworks (see Supplementary Note Supplementary Note 11 for further discussion about the ReAct environment).

Models show the strongest performance in reasoning tasks on the ChemBench corpus, which suggests that current training approaches are good at developing logical/reasoning capabilities for basic problem-solving of university-level exams. However, performance drops significantly for advanced tasks, suggesting that more advanced chemistry problems with complex, multi-step solutions (e.g., solving analytical chemistry problems) should be included in training or finetuning.

The post-training (e.g., reinforcement learning with human feedback (RLHF)) of the current models does not equip them with human-like “chemical taste.” This, however, will be essential for future discovery research (e.g., in combination with genetic algorithms).

Supplementary Note 15 Leaderboard

Our leaderboard is based on the tool chain developed for Matbench.[30] Briefly, the ChemBench pipeline produces standardized files in `json` format that contributors can add via pull requests to the ChemBench repository. The Markdown tables and interactive plots are automatically generated and updated on the ChemBench website. The leaderboard is available at `https://lamalab-org.github.io/chembench/leaderboard/`.

Acronyms

API application programming interface.

CoT chain of thought.

ECE expected calibration error.

GHS globally harmonized system of classification and labelling of chemicals.

IUPAC International Union of Pure and Applied Chemistry.

LLM large language model.

MCQ multiple-choice question.

NMR nuclear magnetic resonance.

REST representational state transfer.

RLHF reinforcement learning with human feedback.

SMILES simplified molecular input line-entry system.

References

1. Schulze Balhorn, L. *et al.* Empirical assessment of ChatGPT's answering capabilities in natural science and engineering. *Sci. Rep.* **14** (Feb. 2024).
2. Microsoft Research AI4Science and Microsoft Azure Quantum. The Impact of Large Language Models on Scientific Discovery: a Preliminary Study using GPT-4. *arXiv preprint arXiv:2311.07361* (2023).
3. Castro Nascimento, C. M. & Pimentel, A. S. Do Large Language Models Understand Chemistry? A Conversation with ChatGPT. *J. Chem. Inf. Model.* **63,** 1649–1655 (Mar. 16, 2023).
4. Northcutt, C. G., Athalye, A. & Mueller, J. Pervasive Label Errors in Test Sets Destabilize Machine Learning Benchmarks. *arXiv preprint arXiv:2103.14749* (2021).
5. Frye, C. *PubMedQA noisy* Dec. 2023. `https://twitter.com/charles%5C_irl/status/1731854677711507650`.

6. Awg. *Broken benchmark: MMLU* 2023. `https://www.lesswrong.com/posts/rQBaftqKMfG2uMiWb/broken-benchmark-mmlu`.
7. Taylor, R. *et al.* Galactica: A Large Language Model for Science. *arXiv preprint arXiv:2211.09085* (2022).
8. Schick, T. *et al.* Toolformer: Language models can teach themselves to use tools. *Adv. Neur. In.* **36** (2024).
9. Karpas, E. *et al.* MRKL Systems: A modular, neuro-symbolic architecture that combines large language models, external knowledge sources and discrete reasoning. *arXiv preprint arXiv:2205.00445* (2022).
10. Yao, S. *et al.* React: Synergizing reasoning and acting in language models. *arXiv preprint arXiv:2210.03629* (2022).
11. Aspuru-Guzik, A., Lindh, R. & Reiher, M. The Matter Simulation (R)evolution. *ACS Cent. Sci.* **4,** 144–152 (Feb. 6, 2018).
12. Guo, T. *et al.* What can Large Language Models do in chemistry? A comprehensive benchmark on eight tasks. *arXiv preprint arXiv:2305.18365* (2023).
13. Sun, L. *et al.* SciEval: A Multi-Level Large Language Model Evaluation Benchmark for Scientific Research. *arXiv preprint arXiv:2308.13149* (2023).
14. Cai, X. *et al.* Comprehensive evaluation of molecule property prediction with ChatGPT. *Methods* **222,** 133–141 (Feb. 2024).
15. Rein, D. *et al.* GPQA: A Graduate-Level Google-Proof Q&A Benchmark. *arXiv preprint arXiv:2311.12022* (2023).
16. Srivastava, A. *et al.* Beyond the Imitation Game: Quantifying and extrapolating the capabilities of language models. *arXiv preprint arXiv:2206.04615* (2022).
17. Yirik, M. A., Sorokina, M. & Steinbeck, C. MAYGEN: an open-source chemical structure generator for constitutional isomers based on the orderly generation principle. *J. Cheminf.* **13** (July 3, 2021).
18. Irwin, J. J., Sterling, T., Mysinger, M. M., Bolstad, E. S. & Coleman, R. G. ZINC: A Free Tool to Discover Chemistry for Biology. *J. Chem. Inf. Model.* **52,** 1757–1768 (June 2012).
19. Actelion. *OpenChemLib* `https://github.com/actelion/openchemlib`.
20. Togo, A., Shinohara, K. & Tanaka, I. `spglib`: a software library for crystal symmetry search. *arXiv preprint arXiv:1808.01590* (2018).
21. Kim, S. *et al.* PubChem 2023 update. *Nucleic Acids Res.* **51,** D1373–D1380 (Oct. 28, 2022).
22. Böttcher, T. An Additive Definition of Molecular Complexity. *J. Chem. Inf. Model.* **56,** 462–470 (Feb. 2016).

23. AI, P. *LLM Guard* https://github.com/protectai/llm-guard.
24. Vercel. *nextjs* https://nextjs.org/.
25. tailwindcss. *Tailwind CSS* https://tailwindcss.com/.
26. NextAuth.js. *NextAuth.js* https://next-auth.js.org/.
27. Wei, J. *et al.* Chain-of-Thought Prompting Elicits Reasoning in Large Language Models. *arXiv preprint arXiv:2201.11903* (2023).
28. Chase, H. *LangChain* Oct. 2022. https://github.com/langchain-ai/langchain.
29. Xiong, M. *et al.* Can LLMs Express Their Uncertainty? An Empirical Evaluation of Confidence Elicitation in LLMs. *arXiv preprint arXiv:2306.13063* (2023).
30. Dunn, A., Wang, Q., Ganose, A., Dopp, D. & Jain, A. Benchmarking materials property prediction methods: the Matbench test set and Automatminer reference algorithm. *npj Comp. Mater.* **6** (Sept. 2020).
